# Choroid Plexus Carcinomas With TP53 Germline Mutations: Management and Outcome

**DOI:** 10.3389/fonc.2021.751784

**Published:** 2021-09-30

**Authors:** Yanong Li, Hailong Liu, Tandy Li, Jin Feng, Yanjiao He, Li Chen, Chunde Li, Xiaoguang Qiu

**Affiliations:** ^1^ Department of Radiation Oncology, Beijing Tiantan Hospital, Capital Medical University, Beijing, China; ^2^ Departments of Pharmacy, New York Presbyterian Lower Manhattan Hospital, New York, NY, United States; ^3^ Department of Pathology, Beijing Tiantan Hospital, Capital Medical University, Beijing, China; ^4^ Department of Neurosurgery, Beijing Tiantan Hospital, Capital Medical University, Beijing, China

**Keywords:** Li-Fraumeni Syndrome (LFS), choroid plexus carcinoma, TP53 germline mutation, radiotherapy, hereditary syndrome in pediatric, pediatric central nervous system tumors

## Abstract

**Background:**

Choroid plexus carcinomas (CPCs) are rare pediatric tumors commonly associated with Li-Fraumeni syndrome (LFS), which involves a germline mutation of the tumor suppressor gene TP53.

**Materials and Methods:**

We retrospectively analyzed the corresponding information of 12 cases, including the effects of surgery and radiotherapy and TP53 germline mutations, to analyse the management strategies. Kaplan-Meier curves and the log-rank test were used to evaluate the progression-free survival (PFS).

**Results:**

Twelve CPC patients were included, of which TP53 germline mutations were found in eight cases. All patients underwent surgical resection, and six patients received radiotherapy following with operation after initial diagnosis, one patient received radiotherapy following relapse. It was significantly different (P=0.012 and 0.028) that patients with TP53 germline mutation receiving the gross total resection (GTR) without radiotherapy showed survival advantages. Without TP53 germline mutations also showed survival advantages, but there is no statistical significance (P=0.063)

**Conclusions:**

These findings provide evidence for the therapeutic strategy that radiotherapy should not be considered for patients with TP53 germline mutations.

## Introduction

Choroid plexus carcinomas (CPCs) are rare malignant tumors in the central nervous system (CNS) with an incidence of only 0.4% (1). According to the 2021 World Health Organization (WHO) classification, CPCs are classified as Grade. III tumors (2). The most common reason for the occurrence of CPCs is related to the dysfunction of the tumor suppressor gene, TP53 (3, 4). It has been reported that TP53 alterations have been observed amongst the 50% of CPC patients (5). Moreover, TP53 germline mutation has been reported to be a rare autosomal dominant disorder, Li-Fraumeni syndrome (LFS) (6). LFS is characterized by multiple primary neoplasms in children and young adults, with a predominance of soft-tissue sarcomas, osteosarcomas, breast cancers, brain tumors and adrenocortical carcinomas (5, 7, 8). Approximately 70%–80% of families with LFS can be detected by performing a follow-up test of TP53 gene mutants (9). As the genome guardian, p53 protein protects cells from various types of DNA damage by activating cell cycle arrest and enables DNA damage mechanisms to repair genome damage. Activation of p53 leads to apoptosis or senescence in the presence of irreversible injury (10). However, dysregulation of p53 protein may allow tumor cells to evade genotoxic signals, including X-rays, bypassing programmed cell death and senescence of the noncanonical pathways (11, 12). Currently, the recommended CPC comprehensive managements include adjuvant radiotherapy after surgical resection. However, based on the molecular function of p53 protein, Michel Bahar et al. (13) proposed that radiotherapy would not improve the survival rate of CPC patients with TP53 gene mutations *via* a meta-analysis. Therefore, in the current study, we retrospectively analyzed the clinicopathological parameters of 12 CPC patients, and their outcomes associated with surgery and radiotherapy and TP53 germline mutations, to evaluate the effects of therapeutic management.

## Materials and Methods

### Study Participates

We collected patients with CPC who had been pathologically confirmed and completed the TP53 gene examination in our institution from 2017 to 2021. Two pathologists reviewed all samples in the Department of Neuropathology in Beijing Neurosurgical Institution, respectively, and reclassified them according to the 2021 WHO classification of CNS tumors. The ethics committee approved the study protocol of the Beijing Neurosurgical Institution.

### Clinicopathological Information

The clinicopathological information included the patient demographics, TP53 mutations, tumor resection, family history, adjuvant chemoradiotherapy and outcomes. Gross total resection (GTR) was defined as no tumor found in postoperative MRI images. Subtotal resection (STR) was established as the resection of 50-95% of masses. Partial resection (PR) was defined as the resection of 10-50% of the mass. The biopsy was performed if less than 10% of the tumor was removed. Tumor progression was defined as tumor relapse or progression of residuals or the discovery of metastasis on the follow-up. Progression-free survival (PFS), defined as the time from random assignment to disease progression or death from any cause (14), and we grouped the patients according to whether the tumor recurred.

### Statistical Analysis

Statistical analysis was performed using SPSS^®^ Statistics 25 (IBM Corp., Armonk, NY). Fisher’s exact test was utilized to evaluate contingency tables. Group comparisons were performed using the Mann-Whitney U test. Progression-free survival (PFS) was defined as the time point from the date of diagnosis to progression, relapse, or the last follow-up. The Kaplan-Meier estimate was performed to analyze the prognosis. The log-rank test was used to compare the survival between the two groups. P < 0.05 indicates a significant correlation.

## Results

**Figure 1:**
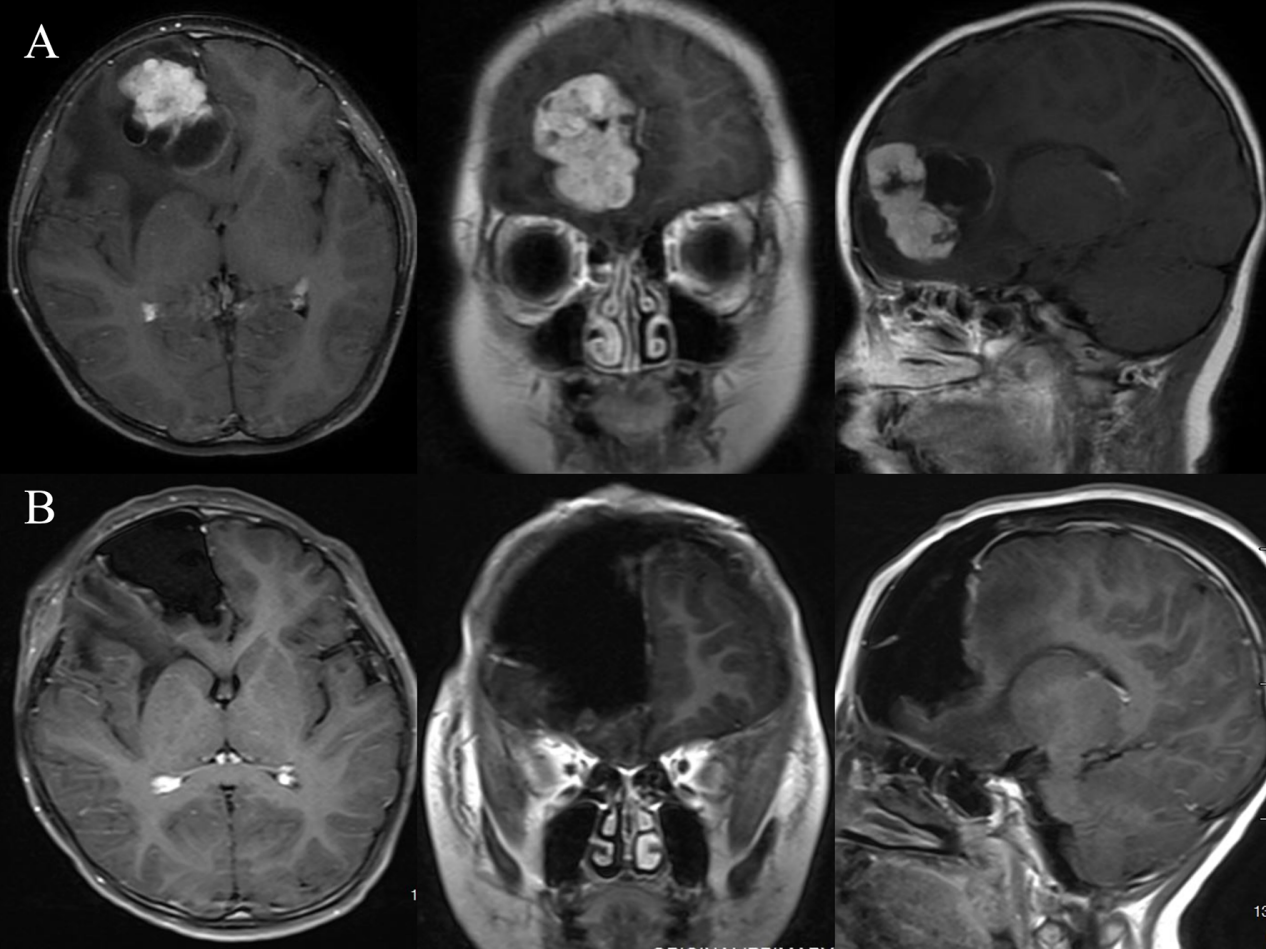
Illustrative case No. 3. A 9-month-old boy presented with headache and vomiting for one week and a contrast-enhanced lesion at the right frontal **(A)**. The tumor was total resected using the right frontal craniotomy approach **(B)**.

**Figure 2:**
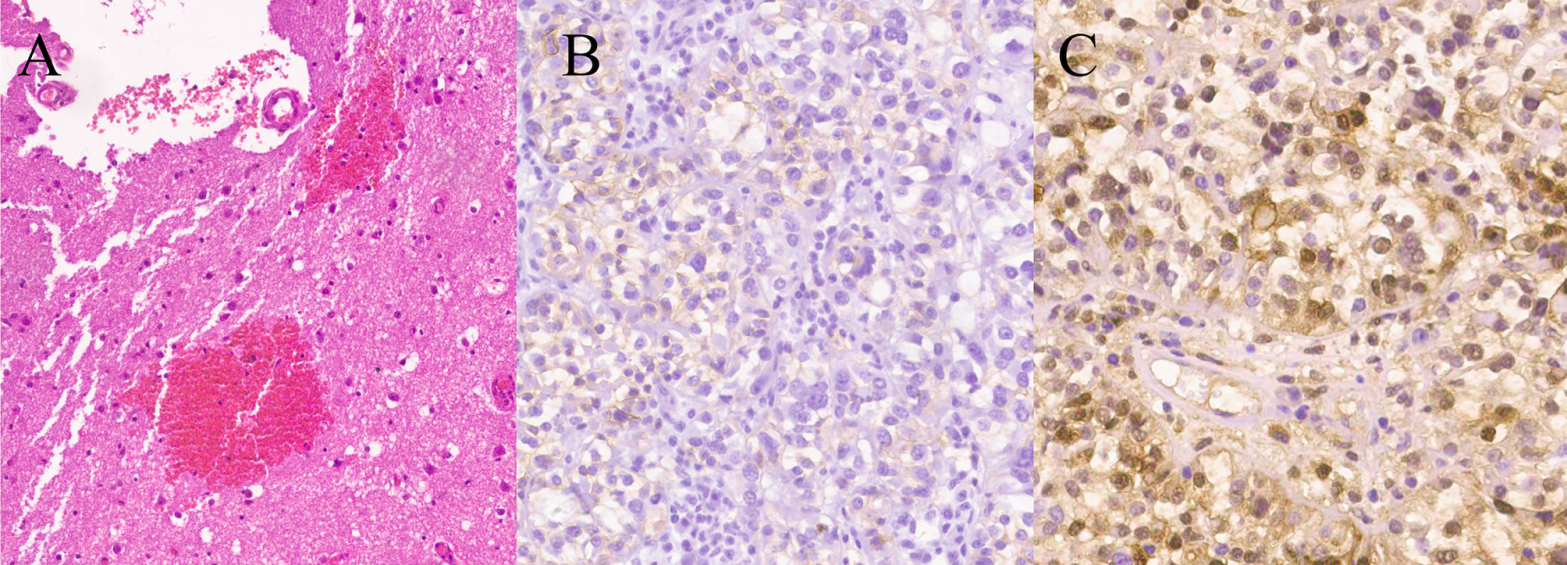
Photomicrographs of surgical specimens. The tumor was well-encapsulated with a distinct fibrous capsule with normal choroid plexus attached (arrow). (H&E stain, ×10) **(A)**. Immunohistochemical staining for CD 99 and S100-beta were positive (×200) **(B, C)**.

**Figure 3:**
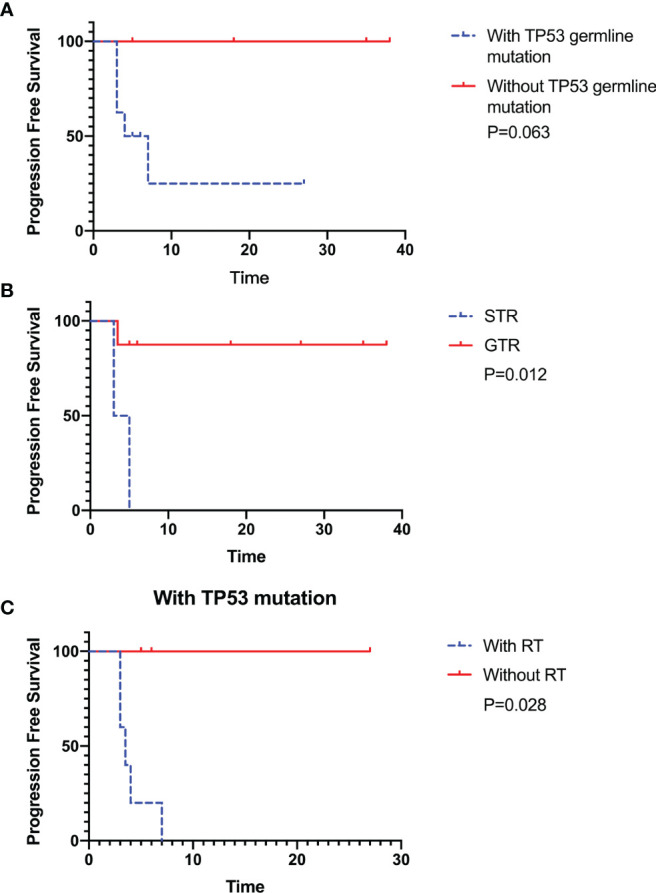
The Kaplan–Meier estimates for progression-free survival (PFS) indicated that patients without TP53 germline mutation showed survival advantages, but there is no statistical significance (n=12, P=0.063) **(A)**. The patients receiving the gross total resection (GTR) and avoiding radiotherapy in patients with TP53 germline mutations showed significant survival advantages (n=12, P=0.012 and n=8, P=0.028) **(B, C)**. All patients were followed up until January 2, 2021.

We collected 12 patients corresponding to the pathologically confirmed CPC with TP53 gene examination. TP53 germline mutations were found in 8 patients, with a male-to-female ratio of 3:1 (9 males and 3 females). The mean age at diagnosis was 66 ± 58.95 months (range 9-231 months) (**Table 1**). The tumor was mainly supratentorial (lateral ventricle in 11 cases; 1 case was in the frontal lobe, and pre- and postoperative MR images were shown in **Figures 1A, B**. The histopathological and immunohistochemical images were shown in **Figures 2A–C**). All patients underwent surgical resection, of which 8 patients with GTR and 4 patients with STR. A total of seven patients received chemotherapy following the operation, including a combination of cisplatin, vincristine, cyclophosphamide, etoposide, and carboplatin. Six patients received postoperative radiotherapy, with a radiotherapy dose of 23.4-24.0 Gy for the whole-brain irradiation (WBI), 30.6-36.0 Gy for craniospinal irradiation (CSI) and 54.0-60.0 Gy for a local tumor or metastatic nodules (4 patients received CSI, and 2 patients received WBI). Five of the 12 patients have relapsed. One patient received radiotherapy after tumor relapse (35.2 Gy craniospinal irradiation was given using 1.8 Gy per fraction and a local boost up to 54.0 Gy). Two of the 12 patients have died, one of whom only received the care after the first surgery and eventually died progression. The other patient suffered from radiation necrosis after 25 fractions of radiotherapy and eventually died of radiation encephalopathy. Of the other ten patients, three experienced relapses, and the others had no evidence of tumor recurrence during the observation period. Patients with STR or TP53 germline mutations had lower survival than those with GTR or without TP53 germline mutations, respectively. In those with TP53 germline mutations, patients treated with radiotherapy had lower survival than those who avoided radiotherapy. The Kaplan-Meier curve showed the survival advantages for patients without TP53 germline mutations (P = 0.063), those who had GTR (P = 0.012), and those who were accompanying TP53 germline mutations but did not receive radiotherapy (P = 0.028) (**Figures 3A–C**).

**Table 1:** Characteristics of clinical and molecular in this study.

No.	Gender	Age (Months)	Surgery	TP53 Germline mutation	dNt point mutation	Amino acid point mutation	Family history	Outcomes (Months)	Radiotherapy (Dose)	CT
1	M	40	STR	N	NA	NA	NA	No evidence of disease (18)	N	Y
2	F	117	GTR	N	NA	NA	NA	No evidence of disease (35)	Y (CSI 36.0 Gy followed by boosts to 60.0 Gy)	Y
3	M	9	GTR	N	NA	NA	NA	No evidence of disease (5)	N	N
4	M	58	GTR	N	NA	NA	NA	No evidence of disease (38)	N	N
5	F	75	STR	Y	c.659A>G	p.Tyr220Cys|p.Y220C	The patient's mother had history of breast cancer and lung cancer	Recurrence (7), dead from disease (24)	Y (CSI 30.6 Gy followed by boosts to 54.0 Gy)	Y
6	M	41	STR	Y	c.818G>A	p.Arg273 His	The maternal aunt died of sarcoma at 25	Recurrence (3)	Y (WBI 23.4 Gy followed by boosts to 54.0 Gy)	Y
7	M	33	GTR	Y	c.1009C>T	p.Arg337 Cys	NA	Recurrence (3)	Y (WBI 24.0 Gy followed by boosts to 54.0 Gy)	Y
8	M	27	GTR	Y	c.524G>A	p.Arg175	NA	Recurrence (4), dead from radioencephalopathy (10)	Y (CSI 30.6 Gy followed by boosts to 54.0 Gy)	N
9	M	41	GTR	Y	c.818G>A	p.Arg273 His	NA	No evidence of disease (5)	N	N
10	F	102	STR	Y	c.916C>T	p.Arg306*|p.R306*	NA	Recurrence (3.5)	Y (CSI 36.0 Gy followed by boosts to 60.0 Gy)	Y
11	M	14	GTR	Y	c.743G>A	p.Arg248 Gln	NA	No evidence of disease (6)	N	Y
12	M	231	GTR	Y	c.4124C>T	p.Ala1375Val|p.A1375V	NA	No evidence of disease (27)	N	N

GTR, gross total resection; STR, subtotal resection; CSI, craniospinal irradiation; WBI, whole brain irradiation; CT, Chemotherapy; NA, Not avalible.

## Discussion

The tumor suppressor TP53 plays a critical role in tumor biology and promotes aggressive tumor behavior. TP53 gene encodes p53 protein, a transcription factor (TF) induced by endogenous and exogenous stresses. These stresses activating signals function *via* post-translational modifications on p53 protein (e.g. phosphorylation, acetylation) leading to p53 activation (15–17). P53 protein eventually enters the nucleus, where it induces the expression of a plethora of target genes (18). Tabori et al. (5) reported that TP53 alterations determined the clinical subgroups and survival of patients with choroid plexus tumors. Approximately 50% of CPC patients harbor TP53 mutations in somatic cells. TP53 mutations in the germline have been associated with higher genomic instability (19–21). Multiple bodies of evidence have supported the association between CPC and TP53 germline mutations. The pediatric CPC cases have a high frequency of TP53 germline mutations in association with LFS (22, 23). In our cohort, patients with TP53 germline mutations receiving the GTR but not radiotherapy showed survival advantages. Based on the abovementioned findings, these signatures could be used to develop a prognostic tool for the targeted treatment for this special subpopulation of children (24, 25). Our study showed that all 8 patients with germline TP53 mutations met the diagnostic criteria of LFS. The PFS of patients with TP53 germline mutations was significantly lower than that of patients without TP53 germline mutations (P = 0.066), indicating the critical role of TP53 dysfunction in determining the response and outcome of treatment amongst these patients. Therefore, for CPC patients, the family and clinical history should be comprehensively collected to verify the LFS. If the information indicates the possibility of LFS, blood samples should be sent for TP53 sequencing and multiple connection-dependent probe amplification analyses (26).

Although surgery is considered the standard initial treatment for CPCs (27), the features of the rich vascular supply and the deep location in lateral ventricles often cause hydrocephalus increasing the difficulty of surgery. Sometimes it is challenging to achieve GTR (28). Is there a need for adjuvant radiotherapy for choroid plexus carcinoma that has been wholly resected? The importance of GTR in the treatment of CPC was confirmed in a retrospective literature review (29). GTR was identified as the single most important predictor of survival. This study analyzed the significant difference of PFS between GTR and STR (P =0.012), consistent with previous research conclusions (19). In addition, Matthew Z Sun et al. (30) carried out a systematic review of the effect of surgery, adjuvant therapy, and other prognostic factors of CPC, which provided evidence of the benefit of GTR in patients with CPC. Our main finding is that GTR significantly improved PFS in the general pediatric CPC population (independent of age, gender, and tumor location), consistent with the other studies (31). Therefore, we strongly recommend invasive excision for the general pediatric CPC population if it can be performed safely.

Several questions remain unanswered regarding the respective benefits and risks of adjuvant radiotherapy in this population. In some earlier retrospective analyses, radiotherapy has been reported to be beneficial for CPC patients. Wolff et al. (13) reported a 5-year survival rate of 68% for the patients receiving radiation therapy compared to 16% for those who did not. However, radiotherapy may increase the risk of secondary tumorigenesis in CPC patients with TP53 mutations in the embryonic line. Bahar et al. (29) conducted a meta-study showing the effect of radiotherapy on 28 patients with LFS. Patients receiving radiotherapy had a lower survival rate than those who did not (18% *vs.* 58% at two years), indicating a possible survival disadvantage in patients with CPC and LFS when treated with radiotherapy. Nevertheless, up to date, it is still under debate that whether radiotherapy could work as a prognostic signature for the CPC patients with TP53 mutation based on the available studies. Therefore, in our cohort of 8 CPC patients with embryogenic TP53 mutation, five patients received radiation therapy, all of whom unfortunately relapsed. Moreover, considering that radiotherapy may increase the risk of secondary tumorigenesis in treated patients, avoiding radiotherapy may improve the PFS of CPC patients with germline TP53 mutations. On the other hand, among patients with mutations analyzed, a part of patients (37.5%) did not undergo total tumor resection, and therefore the increased incidence of recurrence in these patients could also be due to the residual tumor. One patient with TP53 germline mutation died despite the GTR, but he had not undertaken chemotherapy after surgery, so also, in this case, the outcome could have been influenced by other variables and not just by radiotherapy.

The effects of surgery and radiotherapy were evaluated in patients with or without TP53 germline mutations to provide vital information to help define response assessment in neuro-oncology specific to pediatric CPC patients. The study’s main findings showed an improvement in patients receiving tumor total resection and avoiding radiotherapy. In particular, patients with TP53 germline mutation showed a significant survival advantage providing evidence that radiotherapy should not be considered for patients bearing TP53 germline mutation.

This study has some limitations. First, the model of adjuvant radiotherapy and chemotherapy was not unified. In particular, the radio-/chemotherapy strategies for patients were not consistent. Second, the follow-up time was not enough to show the difference in overall survival rate.

## Data Availability Statement 

The datasets presented in this study can be found in online repositories. The names of the repository/repositories and accession number(s) can be found in the article/**Supplementary Material**.

## Ethics Statement

All procedures of this study were conducted according to the Declaration of Helsinki and approved by the Ethics Committee of Beijing Tiantan Hospital. All participants provided written informed consent before entry into the study.

## Author Contributions

YL and HL contributed equally to the paper. All authors contributed to the article and approved the submitted version.

## Funding

This work was supported by the Beijing Municipal Bureau of Health (to XQ, Grant number: 2020-2-1072). The funding sources had no influence on the design, performance, or reporting of this study.

## Conflict of Interest

The authors declare that the research was conducted in the absence of any commercial or financial relationships that could be construed as a potential conflict of interest.

## Publisher’s Note

All claims expressed in this article are solely those of the authors and do not necessarily represent those of their affiliated organizations, or those of the publisher, the editors and the reviewers. Any product that may be evaluated in this article, or claim that may be made by its manufacturer, is not guaranteed or endorsed by the publisher.
