# Supplementary material for: Choroid Plexus Carcinomas With TP53 Germline Mutations: Management and Outcome
Source: Front Oncol. 2021 Sep 30;11:751784. doi: 10.3389/fonc.2021.751784 (PMC8514937; doi:10.3389/fonc.2021.751784)
Supplement: Supplementary file 1 [file DataSheet_1.zip › original_data/Plus_P2003310111_μ¥Äμóôτ║╢_panel825plus_2020.04.27-final 2.pdf]

GENETRON 泛生子

洞 悉 癌 症 全 周 期

肿瘤精准诊疗基因检测
（实体瘤825基因）综合分析报告

ANSWERS
FOR
CANCER

尊敬的李梓纶先生/女士：

首先感谢您对泛生子的信任，选择泛维安 TM 肿瘤精准诊疗基因检测。

肿瘤发生发展的机制非常复杂，即使临床表现相似的受检者，其体内基因变异情况会有很大差别。这种分子水平差异将导致受检者对于同一抗肿瘤治疗的反应不尽相同。肿瘤个体化诊疗是根据受检者肿瘤基因特点，量体裁衣地为其制定最佳的治疗方案，从而提高肿瘤受检者的生存率和生活质量。

随着精准医疗的发展，越来越多的受检者认识到，肿瘤治疗前可通过基因检测，科学地预测药物的疗效和副作用，从而选择适合自己的肿瘤治疗方案。泛维安 TM 肿瘤精准诊疗基因检测通过精确判读实体瘤受检者独有的基因变异信息，为分子分型和预后判断、精准用药及复发监控提供最佳的参考信息。

在战胜肿瘤的道路上，让我们一起披荆斩棘，携手前行！

泛生子全体员工

感谢您选择泛生子的实体瘤 825 基因检测，报告整体分为以下三部分：

第一部分：检测概览

基本信息、检测项目及结果

变异基因结果列表、结果概要

重要基因检测结果及相关解析

第二部分：检测结果详细解析

本次检测匹配到的肿瘤靶向药物、免疫检查点抑制剂和化疗用药提示

本次检测遗传性肿瘤风险提示

检测结果详细解析

第三部分：附录

慈善援助药物信息、已纳入医保的肿瘤药物相关信息

肿瘤精准诊疗实体瘤 825 基因检测列表信息

本次送检样本质控

第一部分：检测概览

一、基本信息

受检者基本信息		样本基本信息	
受检者姓名:	李梓纶	肿瘤样本编号:	F20040231T
性别:	女	肿瘤样本类型:	手术组织石蜡包埋切片
年龄:	2	肿瘤样本采集部位:	未提供
既往诊断结果:	脉络丛乳头状癌	肿瘤样本采集日期:	2020-04-20
既往治疗方案:	无	肿瘤样本接收日期:	2020-04-21
靶向药物服用史:	无	正常样本采集日期:	2020-04-20
癌症家族史:	无	正常样本接收日期:	2020-04-21
		报告日期:	2020-04-27

报告的内容仅适用于专业的科学和医学研究人员进行使用和解读，不包含任何临床建议。
本报告内容可能涉及仍处于临床研究阶段的潜在药物或靶点，这种情况下会明确标出。
Copyright 北京泛生子基因科技有限公司 2020 Ref: Genetron P2003310111

二、检测项目及结果

该产品利用探针杂交捕获技术和 Illumina 高通量测序方法检测 830 个基因的全外显子区和部分内含子区域；检测结果包含覆盖范围内的所有变异类型（点突变、插入缺失突变、拷贝数变异及重排突变），还可提供肿瘤突变负荷（TMB）和微卫星不稳定性（MSI）等分析结果。

检测项目	检测内容	检测意义	检测结果
**体细胞基因变异检测**	830 **个基因点突变、缺失、插入分析**<br>44 **个基因重排分析**<br>88 **个基因拷贝数分析**	**预测靶向药物的有效性**<br>**肿瘤分子分型**<br>**判断预后**	1 **个基因突变**<br>**未见基因重排**<br>**未见基因拷贝数变异**
**肿瘤突变负荷（TMB）**	830 **个基因点突变、缺失、插入分析**	**预测免疫治疗药物疗效**	0.47 **个**/Mb
**微卫星不稳定性（MSI）**	309 **个微卫星位点（**MS**）**	**预测免疫治疗药物疗效**	**微卫星稳定型（**MSS**）**
**化疗单核苷酸多态性（SNP）位点检测**	45 **个** SNP **位点**	**预测常见化疗药物有效性和毒副作用**	**详见检测结果详细解析**
**肿瘤遗传易感基因检测**	148 **个肿瘤遗传易感基因**	**预测多种肿瘤的遗传风险**	**未检测到与肿瘤遗传易感性相关的明确致病突变**

报告的内容仅适用于专业的科学和医学研究人员进行使用和解读，不包含任何临床建议。
本报告内容可能涉及仍处于临床研究阶段的潜在药物或靶点，这种情况下会明确标出。
Copyright 北京泛生子基因科技有限公司 2020 Ref: Genetron P2003310111

三、变异基因结果列表

1、体细胞变异结果

本样本中共检测到 1 个基因的 1 个基因变异。

● 基因点突变、缺失、插入分析结果

基因	突变类型	核苷酸变化	氨基酸变化	氨基酸变化	频率(%)	染色体	外显子	转录本号
TP53	**移码突变**	c.846dup	p.Arg283AlafsTer23	p.R283Afs*23	92.7	17	8/11	NM_001126112.2

● 基因重排分析结果

基因名称	重排基因转录本及断点位置	外显子	变异频率（%）
**未检测出基因重排**			

● 拷贝数分析结果

基因	染色体	拷贝数变异起始位置	拷贝数变异终止位置	变异倍数	变异类型
**未检测出基因拷贝数变异**					

注：1. 根据人类基因组突变学会（HGVS）已建立系统的基因突变命名方法，“c.” 表示编码 DNA 序列，“p.” 表示蛋白序列。

2. 在 DNA 水平对某一突变位点的描述方式包括碱基位点，正常碱基，“>” 符号，突变碱基。

3. 在氨基酸水平，其表示方法是野生型的氨基酸，位点，突变氨基酸，三者之间没有空格。

4. 变异频率：肿瘤样本检测的数据中，支持该基因位点变异的分子数占该位点总分子数的比例。变异频率可能因测序深度和肿瘤取样部位的不同存在差异。

2、胚系变异结果

本样本中未检测到胚系致病变异。

基因	突变类型	核苷酸变化	氨基酸变化	频率(%)	染色体	外显子	转录本号	纯合/杂合	致病风险
**未检测到与肿瘤遗传易感性相关的明确致病突变**									

注：1. 参考基因组版本：GRCh37/hg19；

2. 检测结果过滤标准：保留位于编码区的错义突变、同义突变、无义突变、移码突变以及靠近外显子上下游 1-2bp 之内可变剪切区域的突变；

3. 胚系突变，又叫生殖细胞突变，是来源于精子或卵子这些生殖细胞的突变，通常所有细胞都带有此突变，具有遗传性。

4. 基因变异的致病风险评估依据 ACMG 2015 变异解读指南可分为 1-不致病，2-可能不致病，3-意义不明，4-可能致病，5-致病。本报告仅列出肿瘤遗传相关基因的 5-致病（Pathogenic）或 4-可能致病（Likely Pathogenic）的变异。

报告的内容仅适用于专业的科学和医学研究人员进行使用和解读，不包含任何临床建议。
本报告内容可能涉及仍处于临床研究阶段的潜在药物或靶点，这种情况下会明确标出。
Copyright 北京泛生子基因科技有限公司 2020 Ref: Genetron P2003310111

四、结果摘要

1、靶向治疗药物提示

本次检测匹配到潜在获益的靶向药物分别为：MK-1775。

表 1：潜在获益靶向药物提示表

基因变异	FDA/NMPA 批准于本癌种获益药物或专业临床指南推荐	FDA/NMPA 批准于其它癌种获益药物	本癌种 2 期或 3 期临床试验潜在获益药物	潜在耐药药物
TP53 p.Arg283AlafsTer23	无	无	MK-1775	无

注：1. 本列表中仅给出临床证据等级较高的药物，其它药物参见潜在获益药物列表。
2. FDA：美国食品药品监督管理局；NMPA：国家药品监督管理局。
3. 单个靶点的变异与多靶点药物敏感性的关系目前尚不明确，用药谨请遵医嘱。

2. 免疫治疗药物提示

该样本肿瘤突变负荷（TMB）为 0.47 个/Mb，提示可能从免疫检查点抑制剂单药中获益较小，免疫治疗疗效影响因子较多，需综合评估，用药谨遵医嘱。具体分析结果见第二部分。

该样本微卫星不稳定性（MSI）状态为微卫星稳定型（MSS），提示可能从免疫检查点抑制剂单药中获益较小，免疫治疗疗效影响因子较多，需综合评估，用药谨遵医嘱。具体分析结果见第二部分。

3. 化疗药物提示（仅供参考）

具体分析结果见第二部分。

4. 遗传性肿瘤风险提示

未检测到与肿瘤遗传易感性相关的明确致病突变。具体分析结果见第二部分。

报告的内容仅适用于专业的科学和医学研究人员进行使用和解读，不包含任何临床建议。
本报告内容可能涉及仍处于临床研究阶段的潜在药物或靶点，这种情况下会明确标出。
Copyright 北京泛生子基因科技有限公司 2020 Ref: Genetron P2003310111

五、重要基因检测结果及相关解析

该部分对 FDA 在该癌种获批靶向药物靶点信息以及受检者相关癌种指南中重要基因检测结果和意义小结，包括重要基因的阴性结果及说明：

基因	突变类型	类别	检测结果	解析
IDH1	基因突变	预后	未见突变	*IDH* 和 *TERT* 双突变与 1p19q 联合缺失是少突胶质瘤的分子特征，*IDH* 突变联合 ATRX 缺失是星形细胞瘤的分子特征。*IDH* 突变，预后较好。
IDH2	基因突变	预后	未见突变	*IDH* 和 *TERT* 双突变与 1p19q 联合缺失是少突胶质瘤的分子特征，*IDH* 突变联合 ATRX 缺失是星形细胞瘤的分子特征。*IDH* 突变，预后较好。
ATRX	基因突变	预后	未见突变	*ATRX* 突变、联合 *IDH1/2* 突变以及 1p19q 的状态有助于高级别胶质瘤预后的评估。
CIC	基因突变	诊断、分型	未见突变	*IDH1/2* 突变，1p19q 缺失，*CIC* 突变，分型为少突胶质瘤。*CIC* 突变多见于低级别胶质瘤（少突胶质瘤、毛细胞黏液样胶质瘤）。
EGFR	基因扩增	诊断、分型	未见基因扩增	在 *IDH* 野生型中，*EGFR* 扩增及 EGFRvIII 结构变异提示与胶母细胞瘤相关。原发 GBMs 一般没有 *IDH* 突变，但是有 *EGFR* 扩增和 EGFRvIII 以及 *TERT* 启动子突变。
FUBP1	基因突变	诊断、分型	未见突变	1p/19q 缺失，*IDH1/2*, *CIC*, *FUBP1* 突变，*ATRX* 和 *TP53* 未突变的脑胶质瘤，可分型为少突胶质瘤。位于 19 号染色体上的 *CIC* 基因及 1 号染色体上的 *FUBP1* 基因发生变异可能与少突胶质细胞瘤的形成相关，这两个基因变异很少在其它肿瘤中出现，这意味着 *CIC* 基因和 *FUBP1* 基因是少突胶质细胞瘤的特异基因 。
BRAF	基因突变/基因融合	诊断、分型	未见基因突变/基因融合	*KIAA1549-BRAF* 融合基因和 *BRAF*-Val600Glu 突变与毛细胞型星形细胞瘤密切相关，具有很强的诊断价值;是靶向治疗的标志物。*BRAF* 突变和融合多见于低级别胶质瘤（如 PA,PMA,PXA 和神经节神经胶质瘤)。*BRAF* 和 *KIAA1549* 或 *FAM131B* 的融合，以及 *BRAF*-Val600Glu 突变是毛细胞星形细胞瘤的特征性变异。儿童中，*BRAF* 突变伴随 *CDKN2A* 缺失会增加转化为恶性肿瘤的风险。
H3F3A	p.Lys28Met	诊断、分型	未见突变	与脑干胶质瘤和 GBM 相关。编码组蛋白 H3.3。组织学相似的儿童和成人胶质瘤生物学行为不同，儿童弥漫型胶质瘤明确的基因异常使得一些亚型从组织学相似的成人亚型分开。以组蛋白 H3 基因 *H3F3A* 或更为少见的相关 *HIST1H3B* 基因 K27M 突变为特征的一个狭义的儿童原发肿瘤组（偶见于成人），呈弥漫型生长，且位于中线结构（如丘脑、

报告的内容仅适用于专业的科学和医学研究人员进行使用和解读，不包含任何临床建议。
本报告内容可能涉及仍处于临床研究阶段的潜在药物或靶点，这种情况下会明确标出。
Copyright 北京泛生子基因科技有限公司 2020 Ref: Genetron P2003310111

				脑干和脊髓)。该新定义的组命名为弥漫型中线胶质瘤。
HIST1H3B	基因突变	诊断、分型	未见突变	与脑干胶质瘤和 GBM 相关。编码组蛋白 H3.1。目前检测到儿童 DIPG 特异性较高的分子标记物是编码组蛋白 H3 的基因突变(*H3F3A* 和 *HIST1H3B*)。和儿童脑干胶质瘤的分型相关。儿童脑干胶质瘤中常发生变异，和预后差相关。
HIST1H3C	基因突变	诊断、分型	未见突变	*HIST2H3C* 突变点 K27M 和 *H3F3A* 异亮氨酸取代赖氨酸的突变点 K27I 一样可以导致三甲基化的丢失。和儿童脑干胶质瘤的分型相关。是儿童脑干胶质瘤中常见突变，和预后差相关。
RELA	基因融合	诊断、分型	未见基因融合	70%幕上室管膜瘤中存在 *RELA* 融合，是炎症反应相关基因;融合蛋白会激活 NF-kB 信号通路。室管膜瘤与一些基因融合相关，如 *RELA* 和 *YAP1* 基因。例如，幕上室管膜瘤中常见 *C11orf95/RELA* 融合，且预后较差。
NTRK1/2/3	基因融合	用药	未见基因融合	目前，FDA 批准 Larotrectinib(LOXO-101)用于治疗携带 *NTRK* 基因融合且未携带已知获得性耐药突变的局部晚期或转移性实体瘤的患者。
NAB2	基因融合	诊断、分型	未见基因融合	WHO 中枢神经系统肿瘤分类指南(2016)指出 NAB2-STAT6 融合是“孤立性纤维瘤/血管周细胞瘤”的诊断依据。
SMARCB1	基因突变	诊断、分型	未见突变	WHO 中枢神经系统肿瘤分类指南(2016)指出 SMARCB1 缺失是“非典型畸胎样/横纹肌样瘤”诊断依据和遗传相关分子。
SMARCA4	基因突变	诊断、分型	未见突变	WHO 中枢神经系统肿瘤分类指南(2016)指出 SMARCA4 缺失是“非典型畸胎样/横纹肌样瘤”诊断依据和遗传相关分子。
C19MC	基因融合	诊断、分型	未见基因融合	WHO 中枢神经系统肿瘤分类指南(2016)指出 C19MC 融合是“胚胎性肿瘤伴多层菊形团，C19MC 变异”的诊断依据。
EWSR1	基因融合	诊断、分型	未见基因融合	WHO 中枢神经系统肿瘤分类指南(2016)指出 EWSR1 融合是“尤文肉瘤/外周原始外胚层肿瘤”的诊断依据。

报告的内容仅适用于专业的科学和医学研究人员进行使用和解读，不包含任何临床建议。
本报告内容可能涉及仍处于临床研究阶段的潜在药物或靶点，这种情况下会明确标出。
Copyright 北京泛生子基因科技有限公司 2020 Ref: Genetron P2003310111

第二部分：检测结果详细解析

一、肿瘤靶向治疗药物用药提示

1. 潜在获益靶向药物提示

本次检测匹配到潜在获益的靶向药物，具体见第一部分中的结果摘要-靶向治疗药物提示。

2. 临床意义未明靶向药物提示

本次检测未匹配到临床意义未明靶向药物。

二、肿瘤免疫检查点抑制剂用药提示

1.肿瘤突变负荷（TMB）分析结果提示

该样本肿瘤突变负荷（TMB）为 0.47 个/Mb，提示可能从免疫检查点抑制剂单药中获益较小，免疫治疗疗效影响因子较多，需综合评估，用药谨遵医嘱。

检测结果	0.47 个/Mb
结果解析	肿瘤突变负荷（TMB：Tumor Mutation Burden），表示一份肿瘤样本中，基因组上的外显子编码区每 Mb 碱基中发生置换、插入、缺失的体细胞突变总数。已有临床试验表明，具有较高水平的 TMB 肿瘤细胞更容易被免疫系统识别，因此能对免疫检查点抑制剂有更强的免疫应答。如肿瘤突变负荷越大，对免疫检查点抑制剂 Nivolumab，Pembrolizumab，Atezolizumab 等单药可能会有较好应答[ASCO 2017, Abstract 3039; ASCO 2017, Abstract e14529; PMID:28636851; PMID:27694933]。TMB≥10 时，提示肿瘤突变负荷较高，但 TMB 高低与检测 panel 大小、基因选择、样本类型、癌种类型等因素有关，检测结果仅供参考。

注：免疫治疗疗效影响因素较多，不是高突变负荷或 MSI-H 免疫治疗一定有效，低突变负荷或 MSS 免疫治疗一定无效，用药请谨遵医嘱。

报告的内容仅适用于专业的科学和医学研究人员进行使用和解读，不包含任何临床建议。
本报告内容可能涉及仍处于临床研究阶段的潜在药物或靶点，这种情况下会明确标出。
Copyright 北京泛生子基因科技有限公司 2020 Ref: Genetron P2003310111

2.微卫星不稳定分析结果提示

该样本微卫星不稳定性（MSI）状态为微卫星稳定型（MSS），提示可能从免疫检查点抑制剂单药中获益较小，免疫治疗疗效影响因子较多，需综合评估，用药谨遵医嘱。

检测结果	微卫星稳定型（MSS）
结果解析	微卫星不稳定（MSI：microsatellite instability）是指 DNA 序列中简单重复序列的碱基长度和（或）重复次数的增加或减少，产生遗传不稳定性。本检测利用 NGS 的方法评估 309 个微卫星位点（MS）长度分布来判断 MSI 状态，仅供参考。<br>有研究表明，存在微卫星不稳定的肿瘤患者，更倾向于从 PD-1 类抑制剂治疗中获益 [PMID:26028255]，如 Pembrolizumab 和 Nivolumab。

注： 免疫治疗疗效影响因素较多，不是 MSI-H 或高突变负荷免疫治疗一定有效，MSS 或低突变负荷免疫治疗一定无效，用药谨请遵医嘱。

3. 其它可能影响免疫治疗疗效的基因检测结果

实体瘤 825 基因检测包含与免疫治疗相关基因：正向基因和负向基因，该基因来源于某些癌种的初步研究成果，结果仅供参考。

- 实体瘤：

基因	免疫治疗相关性	检测结果	位点解析	检测意义
MLH1	正向基因	未见突变	\	FDA 批准 Pembrolizumab 用于 MSI-H（或 dMMR）的实体瘤患者，批准 Nivolumab 用于不可切除或转移性的 MSI-H (或 dMMR)的结直肠癌患者。
MSH2	正向基因	未见突变	\	
MSH6	正向基因	未见突变	\	
PMS2	正向基因	未见突变	\	
POLE	正向基因	未见突变	\	在子宫内膜癌中，*POLE* 突变与高突变负荷、PD-1 和 PD-L1 表达增加、T 细胞和淋巴细胞浸润相关 [PMID:25394778; PMID:25878334; PMID:25931171; ASCO 2015, Abstract 5511; PMID:23636398]，表明携带 *POLE* 突变的肿瘤能够从免疫检查点抑制剂治疗中获益 [PMID:27159395; PMID:29163851]。
POLD1	正向基因	未见突变	\	POLD1 在调节细胞周期进程和 DNA 损伤修复中起重要作用 [PMID:26087769; PMID:27974823]。携带 *POLD1* 突变的结直肠癌往往具有微卫星稳定而染色体不稳定的特点 [PMID:23263490],提示携带 *POLD1* 突变的肿瘤能够从免疫检查点抑制剂治疗中获益。

报告的内容仅适用于专业的科学和医学研究人员进行使用和解读，不包含任何临床建议。
本报告内容可能涉及仍处于临床研究阶段的潜在药物或靶点，这种情况下会明确标出。
Copyright 北京泛生子基因科技有限公司 2020 Ref: Genetron P2003310111

DDR gene	正向基因	未见突变	\	DDR 基因突变导致 TMB 值升高、肿瘤浸润淋巴细胞增加，携带 DDR 基因突变的患者更有可能从免疫治疗中获益。
MDM2	负向基因	无扩增	\	研究报道携带 *MDM2/4* 扩增的实体瘤患者可能使用抗 PD-L1 的药物疾病出现爆发式进展[PMID:28351930; EMSO 2017,1140PD; ASCO 2019, Abstract 2557]。
MDM4	负向基因	无扩增	\	
CCND1	负向基因	无扩增	\	*CCND1*、*FGF3*、*FGF4* 和 *FGF19* 均位于染色体 11q13，上述基因的拷贝数变异可能与免疫检查点抑制剂（ICIs）治疗引起的超进展有关[Annals of Oncology, 2017, 28(suppl_5)]。
FGF19	负向基因	无扩增	\	
FGF3	负向基因	无扩增	\	
FGF4	负向基因	无扩增	\	
B2M	负向基因	未见突变	\	*B2M* 基因缺失突变的非炎性肿瘤较不容易被细胞毒性 T 细胞浸润，临床数据显示 *B2M* 缺失可能是黑色素瘤患者对常见的靶向 CTLA4 或 PD1 治疗药物的耐药机制[PMID:29070816; PMID:30294272]。

注：此部分 DDR gene 不包含 *POLE* 和 *POLD1*。

- 胶质母细胞瘤：

基因	免疫治疗相关性	检测结果	位点解析	检测意义
BRAF	正向基因	未见突变	\	2019 年《Nature Medicine》发表的研究纵向分析使用 PD-1 抑制剂的 66 位复发胶质母细胞瘤患者，其中包括 17 位长期生存的患者。研究发现治疗响应组主要富集了 *BRAF* 基因和 *PTPN11* 基因（p=0.018），未响应组主要富集了 *PTEN* 基因（p=0.0063）[PMID:30742119]。
PTPN11	正向基因	未见突变	\	
PTEN	负向基因	未见突变	\	

- 非小细胞肺癌：

基因	免疫治疗相关性	检测结果	位点解析	检测意义
EGFR	负向基因	未见突变	\	CheckMate-057、Keynote-010、POPLAR 临床试验的亚组分析及一项 meta 分析研究显示，既往接受过至少一线治疗的、携带 EGFR 激活突变与携带 *EGFR* 野生型非小细胞肺癌患者相比，从 PD-L1 免疫治疗中获益概率较低，原因可能为 PD-L1 表达在 *EGFR* 突变型患者中较低[PMID:26712084; PMID:26412456; PMID:26970723; PMID:27765535]，然而在一项 Atezolizumab 作为一线或后续治疗的 2 期 BIRCH 临床试验研究显示，在未接受过既往治疗

报告的内容仅适用于专业的科学和医学研究人员进行使用和解读，不包含任何临床建议。
本报告内容可能涉及仍处于临床研究阶段的潜在药物或靶点，这种情况下会明确标出。
Copyright 北京泛生子基因科技有限公司 2020 Ref: Genetron P2003310111

				的非小细胞肺癌患者中，*EGFR* 野生型和突变型的患者间未显示出生存差异[PMID:28609226]。
ALK	负向基因	未见突变	\	多项临床研究表明，无论 PD-L1 表达水平如何，免疫疗法在携带驱动基因突变如 *EGFR*、*ALK* 基因重排的 NSCLC 患者中表现欠佳[PMID:26412456; PMID:26028407; PMID:26712084; PMID:27225694]。
MET	负向基因	未见突变	\	有临床研究显示，*MET* 基因 14 号外显子变异且 PD-L1 高表达的 NSCLC 患者与未筛选的患者相比，中位 TMB 较低。这类患者接受 PD1 抑制剂治疗偶有响应，但是总体临床疗效并不理想[PMID:30165371]。有案例报道称，1 名 *EGFR* 和 *ALK* 阴性，PD-L1 高表达（TPS=95%），*MET* EX14 跳跃突变的 71 岁无吸烟史的女性非小细胞肺癌患者接受帕博利珠单抗一线治疗，但无临床获益 [PMID:30600919]。
KRAS	正向基因	未见突变	\	有研究发现，携带 *KRAS/TP53* 突变的肺肿瘤中，PD-L1 的表达显著提高，T 细胞的浸润性和肿瘤免疫敏感性增强，同时，研究人员也发现，携带 *TP53*、*TP53/KRAS*、*KRAS* 突变的患者能获益于 PD-1 抑制剂，因而，携带 *KRAS/TP53* 突变提示患者可能获益于 PD-L1/PD-1 的治疗，但不同癌种中患者获益的 OS 差异较大[PMID:28039262; PMID:29572005; PMID:29339375; PMID:29484144]。
TP53	正向基因	p.Arg283AlafsTer23	失活突变，突变对蛋白功能可能有影响	
STK11	负向基因	未见突变	\	*STK11* 的失活与“冷”的肿瘤免疫微环境有关[PMID:26833127; PMID:19629071]，*STK11* 失活突变肿瘤中 PD-1 配体 PD-L1 的表达降低，PD-1 靶向抗体对 STK11 缺陷肿瘤通常无效[PMID:19629071]。有肺癌的研究报道[PMID:29773717]，存在 *KRAS/STK11* 共突变的患者接受 PD-1/PD-L1 抑制剂治疗的 PFS 及 OS 要显著低于 *KRAS* 突变-*STK11* 野生类型患者。

- 黑色素瘤：

基因	免疫治疗相关性	检测结果	位点解析	检测意义
JAK1	负向基因	未见突变	\	在黑色素瘤的案例中发现，*JAK1/2* 的失活突变可导致 PD-1 阻断治疗耐药。干扰素 γ（Interferon-γ）是肿瘤微环境中重要的炎性因子，可通过 JAK1/2-STAT1 通路引导其他具有免疫效应功能的基因的表达，并且可诱导 PD-L1 的表达 [PMID:29921905]。因此，*JAK1/2* 的杂
JAK2	负向基因	未见突变	\	

报告的内容仅适用于专业的科学和医学研究人员进行使用和解读，不包含任何临床建议。
本报告内容可能涉及仍处于临床研究阶段的潜在药物或靶点，这种情况下会明确标出。
Copyright 北京泛生子基因科技有限公司 2020 Ref: Genetron P2003310111

				合性缺失突变可引起干扰素 γ 信号通路受损，进而导致 PD-L1 表达下调，使肿瘤细胞对 PD-1 阻断治疗无响应。此外，研究推断，在 *JAK1/2* 失活情况下，抗肿瘤 T 细胞识别及杀死癌细胞的能力下降 [PMID:27903500]。
CDKN2A	正向基因	未见突变	\	*CDKN2A* 基因突变的患者易发生较高的突变负荷，大部分的患者表达 PD-L1[ASCO 2018,Abstract 9102]。据报道，*CDKN2A* 突变的恶性黑色素瘤患者接受一线免疫治疗后，有较好的预后效果[ASCO 2017,Abstract e21057]。*CDKN2A* 基因和 *JAK2* 基因定位于染色体 9p 上临近的位置，两者共缺失时，对免疫类治疗产生耐药性 [PMID:29917141; ESMO 2018,PO-399]。
PTEN	负向基因	未见突变	\	*PTEN* 基因缺失会激活 PI3K-AKT 通路，形成肿瘤。据报道，*PTEN* 突变抑制 T 细胞介导的肿瘤细胞凋亡，产生 PI3K/Akt/mTOR 通路的耐药表型，在黑色素瘤中会增强肿瘤对免疫药物类的耐药性[PMID:26645196]。
DNMT3A	负向基因	未见突变	\	携带 *DNMT3A* 突变的晚期黑色素瘤患者，接受 PD-1 抑制剂治疗后，容易发生爆发式进展 [PMID:28351930]。
NRAS	正向基因	未见突变	\	有临床研究发现，*NRAS* 突变的黑色素瘤患者 PD-L1 表达相对较高。该项回顾性分析结果显示，携带 *NRAS* 突变的晚期黑色素瘤患者从免疫为基础的治疗中获益增加[PMID:25736262]。
SERPINB3	正向基因	未见突变	\	*SERPINB3/4* 突变能够增进肿瘤新抗原呈递。一项临床研究结果提示，携带 *SERPINB3/4* 突变的黑色素瘤患者使用 CTLA4 抗体治疗可以获得较好的获益[PMID:27668655]。在 CA209-038 临床研究中，入组 68 名既往接受或未接受过 Ipilimumab 治疗进展的黑色素瘤患者，接受纳武利尤单抗（Nivolumab）的治疗，6 名携带 *SERPINB3/4* 突变的患者中有 5 名疾病得到了控制（CR/PR 或 SD），但由于样本量较小，未发现单个基因变化与治疗显著相关的统计学意义 [PMID:29033130]。
SERPINB4	正向基因	未见突变	\	
APLNR	负向基因	未见突变	\	小鼠模型中 *APLNR* 基因功能缺失突变降低过继细胞转移疗效，影响免疫检查点抑制剂的抗肿瘤效果[PMID:28783722]。
IFNGR1	负向基因	未见突变	\	干扰素 γ（Interferon-γ）是肿瘤微环境中重要的炎性因子，其受体由 IFNGR1 和 IFNGR2 两个亚单位组成，可通过 JAK1/2-STAT1 通路引导其他具有免疫效应功能的基因的表达，并且可诱导 PD-L1、CTLA-4 等免疫逃逸相关分子的表达，
IFNGR2	负向基因	未见突变	\	

报告的内容仅适用于专业的科学和医学研究人员进行使用和解读，不包含任何临床建议。
本报告内容可能涉及仍处于临床研究阶段的潜在药物或靶点，这种情况下会明确标出。
Copyright 北京泛生子基因科技有限公司 2020 Ref: Genetron P2003310111

				IFN γ 通路受损可引起肿瘤细胞对免疫治疗耐药[PMID:30039553]。有报道 *IFNGR1* 基因敲除的黑色素瘤细胞可引起 IFN γ 信号缺失，并对抗 CTLA-4 治疗耐药 [PMID:27667683]。IFN γ 通路相关基因如 *IFNGR1*、*IFNGR2* 缺失的黑色素瘤细胞可促使对免疫治疗的耐药[PMID:28723893]。
CTNNB1	负向基因	未见突变	\	*CTNNB1* 基因（编码 β-catenin 蛋白）的激活突变导致 WNT/β-catenin 信号通路的异常激活。有研究指出，携带 *CTNNB1* 基因功能获得性突变的黑色素瘤细胞中 T 细胞为非炎症表型。小鼠模型实验结果进一步提示，肿瘤细胞中 β-catenin 通路内源性激活导致 T 细胞排斥，引起抗 PD-L1 或抗 CTLA-4 单克隆抗体治疗的耐药[PMID:25970248]。

- 肾癌：

基因	免疫治疗相关性	检测结果	位点解析	检测意义
PBRM1	正向基因	未见突变	\	临床研究指出，*PBRM1* 基因缺失或截断与免疫相关基因如 IL6/JAK-STAT3 信号通路基因表达升高有关，而临床数据显示与 *PBRM1* 野生型相比，携带 *PBRM1* 截断突变的肾透明细胞癌患者接受纳武利尤单抗或阿特珠单抗治疗的临床获益明显较高[PMID:29301960]。

注：1、上述免疫治疗相关基因是指既往研究支持的可能与免疫治疗疗效有关的变异基因，但患者检测到的变异可能与文献报道的基因变异不完全一致，部分基因变异的致病性和临床意义不明或缺乏功能验证结果，请结合其它检测结果和临床资料谨慎用药。

2、正向基因：与免疫治疗疗效正相关的基因；负向基因：与免疫治疗疗效负相关的基因。

报告的内容仅适用于专业的科学和医学研究人员进行使用和解读，不包含任何临床建议。
本报告内容可能涉及仍处于临床研究阶段的潜在药物或靶点，这种情况下会明确标出。
Copyright 北京泛生子基因科技有限公司 2020 Ref: Genetron P2003310111

三、化疗药物用药提示（仅供参考）

该部分为根据受检者的单核酸多态性（SNP）突变位点结合相关数据库，得出的关于受检者化疗药物有效性和毒副作用风险的用药提示，并且化疗药物的使用与受检者身体情况、既往治疗等情况密切相关，该内容仅供参考，用药请谨遵医嘱。

药物名称	基因	检测位点	检测结果	类别	证据等级	用药提示
氟尿嘧啶类药物为基础的化疗方案	*DPYD*	rs2297595	TT 野生型	药物毒副作用	Level 3	携带 TT 型基因的肿瘤患者，相比于 CC 和 CT 基因型，可能有（1）毒副反应风险减小；（2）药物代谢率升高；然而目前也有不一致性研究结论。
氟尿嘧啶类药物为基础的化疗方案	*DPYD*	rs3918290	CC 野生型	药物有效性	Level 3	携带 CC 基因型的肿瘤患者，相比于 CT 基因型，可能有较高的药物缓解率。然而，也有结论不一致的研究。
氟尿嘧啶类药物为基础的化疗方案	*DPYD*	rs17376848	AA 野生型	药物毒副作用	Level 3	携带 AA 基因型的肿瘤患者，相比 AG 或 GG 基因型，可能有较低的药物毒性风险。然而，也有研究发现药物毒性与基因型无相关性。
氟尿嘧啶类药物为基础的化疗方案	*DPYD*	rs1801158	CC 野生型	药物毒副作用	Level 3	携带 CC 基因型的肿瘤患者，相比 CT 基因型，1）可能有较低的药物毒性风险；2）可能有较高的 DPYD 活性。然而，也有研究发现药物毒性和 DPYD 活性与基因型无相关性。
氟尿嘧啶类药物为基础的化疗方案	*DPYD*	rs1801159	TT 野生型	药物毒副作用/有效性	Level 3	携带 TT 基因型的肿瘤患者，相比 CT 或 CC 基因型，1）可能有较低的恶心呕吐和白细胞减少症发生风险；2）可能有较高的药物缓解率；3）可能有较高的氟尿嘧啶清除率。然而，也有研究发现药物毒性与基因型无相关性或有相反的相关性。
氟尿嘧啶类药物为基础的化疗方案	*DPYD*	rs1801160	CC 野生型	药物毒副作用	Level 3	携带 CC 基因型的肿瘤患者，相比 CT 或 TT 基因型，1）可能有较高的药物代谢；2）可能有较低的药物毒性风险。
氟尿嘧啶类药物为基础的化疗方案	*DPYD*	rs56038477	CC 野生型	药物毒副作用	Level 3	携带 CC 基因型的肿瘤患者，相比 CT 或 TT 基因型，可能有较低的药物毒性发生风险。
氟尿嘧啶+亚叶酸+奥沙利铂	*ERCC1*	rs11615	AG 杂合型突变	药物毒副作用	Level 3	携带 AG 基因型的结直肠癌患者，相比于 AA 基因型，嗜中性白血球减少症风险可能减小。

报告的内容仅适用于专业的科学和医学研究人员进行使用和解读，不包含任何临床建议。
本报告内容可能涉及仍处于临床研究阶段的潜在药物或靶点，这种情况下会明确标出。
Copyright 北京泛生子基因科技有限公司 2020 Ref: Genetron P2003310111

氟尿嘧啶+亚叶酸+奥沙利铂	*ERCC1*	rs11615	AG 杂合型突变	药物有效性	Level 3	携带 AG 基因型的结直肠癌患者，相比于 GG 基因型，总生存期和无进展生存期可能缩短。
氟尿嘧啶+亚叶酸+奥沙利铂	*MTHFR*	rs1801133	AA 纯合型突变	药物毒副作用/有效性	Level 3	携带 AA 基因型的结肠肿瘤患者，相比于 AG 和 GG 基因型，可能有较低的药物缓解率和较高毒性风险。然而，也有结论不一致研究。
氟尿嘧啶+亚叶酸+奥沙利铂	*XRCC1*	rs25487	CT 杂合型突变	药物有效性	Level 3	携带 CT 基因型的患者，相比于 CC 基因型，可能有较差的药物缓解率；感觉神经的病变风险增加或可能早发。
氟尿嘧啶+亚叶酸+奥沙利铂	*ERCC2*	rs13181	TT 野生型	药物毒副作用/有效性	Level 3	携带 TT 基因型的结直肠癌患者，相比 GG 基因型，1）可能有较低的药物毒性风险；2）可能有较低的早期复发风险；3）可能较高的无进展生存期。
氟尿嘧啶	*GSTP1*	rs1695	AA 野生型	药物毒副作用	Level 3	携带 AA 基因型的直肠癌患者，相比于 GG 基因型，血液毒性风险可能增加。
氟尿嘧啶	*DPYD*	rs115232898	TT 野生型	药物毒副作用	Level 3	携带 TT 基因型的患者，相比 CT 基因型，可能有较低的药物毒性风险。
氟尿嘧啶	*DPYD*	rs1801265	AA 纯合型突变	药物毒副作用	Level 3	携带 AA 基因型的肿瘤患者，相比 AG 或 GG 基因型，1）可能有较低的药物毒性风险；2）可能有较高的药物缓解率；3）可能有较高的氟尿嘧啶清除率；4）可能有较低的 DPYD 活性。然而，也有研究发现药物毒性有相反的相关性。
氟尿嘧啶	*EGFR*	rs2293347	CC 野生型	药物有效性	Level 3	携带 CC 基因型的胃癌患者，相比 CT 或 TT 基因型，可能有较低的药物缓解率。
卡培他滨	*ABCB1*	rs1045642	AG 杂合型突变	药物毒副作用	Level 3	携带 AG 基因型的结直肠癌患者，相比于 AA 基因型，可能有较高的手足综合征发病风险。
卡培他滨	*CDA*	rs2072671	AA 野生型	药物毒副作用	Level 3	携带 AA 基因型的肿瘤患者，相比于 AC 或 CC 基因型，可能有较高的毒副风险，包括手足综合征。然而目前有结论不一致性研究。
伊立替康	*UGT1A1*	rs8175347	(TA)6/(TA)6 野生型	药物毒副作用	Level 2A	携带(TA)6/(TA)6 基因型的肿瘤患者，相比于 (TA)6/(TA)7 和 (TA)7/(TA)7 基因型，毒副反应风险可能减小（包括嗜中性白血球减少症、腹泻、乏力）。

报告的内容仅适用于专业的科学和医学研究人员进行使用和解读，不包含任何临床建议。
本报告内容可能涉及仍处于临床研究阶段的潜在药物或靶点，这种情况下会明确标出。
Copyright 北京泛生子基因科技有限公司 2020 Ref: Genetron P2003310111

伊立替康	*UGT1A1*	rs8175347	(TA)6/(TA)6 野生型	药物剂量	Level 3	携带(TA)6/(TA)6 基因型的肿瘤患者，使用伊立替康为基础的治疗，正常剂量使用。
奥沙利铂	*GSTP1*	rs1695	AA 野生型	药物毒副作用	Level 3	携带 AA 基因型的结直肠癌患者，相比于 AG 和 GG 基因型，神经毒性和不耐受停药的风险可能增加。携带 AA 基因型的消化道肿瘤患者，相比于 AG 和 GG 基因型，可能有较高的周围神经系统疾病风险。
顺铂	*GSTP1*	rs1695	AA 野生型	药物毒副作用	Level 2B	携带 AA 基因型的患者，相比于 AG 和 GG 基因型，耳毒性的风险可能降低。
卡铂	*MTHFR*	rs1801133	AA 纯合型突变	药物有效性	Level 2A	携带 AA 基因型的肺癌或者间皮瘤患者，相比于 AG 和 GG 基因型，可能有较好的药物缓解率和较长的无进展生存期。
顺铂/卡铂/奥沙利铂/铂类化合物	*ERCC1*	rs11615	AG 杂合型突变	药物毒副作用/有效性	Level 2B	携带 AG 基因型的患者，相比于 GG 基因型，可能有较高中毒性肾损伤的风险、较低的生存率和较低的药物缓解率。然而这种相关性还有争议。
顺铂/卡铂/奥沙利铂/铂类化合物	*XRCC1*	rs25487	CT 杂合型突变	药物有效性	Level 2B	携带 CT 基因型的肿瘤患者，相比于 CC 基因型，（1）可能有较低的生存率及药物缓解率；（2）严重中性粒细胞减少症风险降低。
顺铂/卡铂/奥沙利铂/铂类化合物	*ERCC1*	rs3212986	AC 杂合型突变	药物有效性	Level 3	携带 AC 基因型的肺癌、食管癌和卵巢癌患者，相比于 CC 基因型，可能有较高的总体生存率。
铂类化合物	*ERCC2*	rs1052555	GG 野生型	药物有效性	Level 3	携带 GG 基因型的非小细胞肺癌患者，相比于 AA 或 AG 基因型，可能有较高的药物缓解率。
铂类化合物	*ERCC2*	rs13181	TT 野生型	药物毒副作用	Level 3	携带 TT 基因型的非小细胞肺癌患者，相比 GG 基因型，可能有较低的肺炎发生风险。
铂类化合物	*VEGFA*	rs25648	CC 野生型	药物有效性	Level 3	携带 CC 基因型的转移性胃癌患者，相比 CT 或 TT 基因型，可能有较高的药物缓解率。
铂类化合物+紫杉醇类	*GSTP1*	rs1695	AA 野生型	药物毒副作用	Level 3	携带 AA 基因型的患者，相比于 AG 和 GG 基因型，血液毒性、神经毒性、中性粒细胞减少和中止治疗的风险可能增加。
顺铂+环磷酰胺	*NAT2*	rs1801280	TT 野生型	药物毒副作用	Level 3	携带 TT 基因型的卵巢癌患者，相比 CT 基因型，可能有较低的 2-4 级贫

报告的内容仅适用于专业的科学和医学研究人员进行使用和解读，不包含任何临床建议。
本报告内容可能涉及仍处于临床研究阶段的潜在药物或靶点，这种情况下会明确标出。
Copyright 北京泛生子基因科技有限公司 2020 Ref: Genetron P2003310111

						血症发生风险。
卡铂+多西他赛+曲妥珠单抗	*ERBB2*	rs1136201	AA 野生型	药物有效性	Level 3	携带 AA 基因型的乳腺癌患者，相比于 AG 或 GG 基因型，可能有较高的药物缓解率。
卡铂+多西他赛+曲妥珠单抗	*ERBB3*	rs2229046	TT 野生型	药物有效性	Level 3	携带 TT 基因型的乳腺癌患者，相比 CC 或 CT 基因型，可能有较高的药物缓解率。
卡铂+多西他赛+曲妥珠单抗	*ERBB3*	rs773123	AA 野生型	药物有效性	Level 3	携带 AA 基因型的乳腺癌患者，相比 TT 基因型，可能有较低的药物缓解率。
吉西他滨	*CDA*	rs2072671	AA 野生型	药物毒副作用	Level 3	携带 AA 基因型的肿瘤患者，相比于 CC 基因型，（1）可能有胞苷脱氨酶（CDA）表达降低（2）可能有较高的毒副风险，比如嗜中性白血球减少症和胃肠道毒性。然而目前有结论不一致性研究。
吉西他滨	*CDA*	rs60369023	GG 野生型	药物毒副作用	Level 3	携带 GG 基因型的肿瘤患者，相比于 AA 基因型，（1）可能有较高的吉西他滨清除，（2）可能有较低的嗜中性白血球减少症风险。
培美曲塞	*GGH*	rs11545078	GG 野生型	药物毒副作用	Level 3	携带 GG 基因型的肺癌患者，相比于 AA 基因型，可能有较高的药物毒副风险。
培美曲塞	*MTHFR*	rs1801133	AA 纯合型突变	药物有效性	Level 3	携带 AA 基因型的肺癌或者间皮瘤患者，相比于 GG 基因型，可能有较短的总生存期。
培美曲塞	*DHFR*	rs442767	TT 纯合型突变	药物毒副作用	Level 3	携带 TT 基因型的肺癌患者，相比 GG 基因型，可能有较低的疲劳风险。
环磷酰胺	*MTHFR*	rs1801133	AA 纯合型突变	药物毒副作用	Level 2A	携带 AA 基因型的肿瘤患者，相比于 AG 和 GG 基因型，可能有较高的药物毒副风险。
环磷酰胺	*XRCC1*	rs25487	CT 杂合型突变	药物毒副作用/有效性	Level 3	携带 CT 基因型的卵巢癌患者，相比于 CC 基因型，可能有较低发生严重嗜中性白血球减少症的风险，然而可能有较短的无进展生存期和总生存期。
环磷酰胺+阿霉素	*SLC22A16*	rs12210538	AA 野生型	药物毒副作用	Level 3	携带 AA 基因型的女性乳腺癌患者，相比于 GG 基因型，可能有较低的药物毒副风险。
环磷酰胺+阿霉素	*CYP2B6*	rs3211371	CC 野生型	药物毒副作用	Level 3	携带 CC 基因型的女性乳腺癌患者，相比于 CT 和 TT 基因型，可能有较低的剂量延迟反应风险。白细胞减

报告的内容仅适用于专业的科学和医学研究人员进行使用和解读，不包含任何临床建议。
本报告内容可能涉及仍处于临床研究阶段的潜在药物或靶点，这种情况下会明确标出。
Copyright 北京泛生子基因科技有限公司 2020 Ref: Genetron P2003310111

						少症及嗜中性白血球减少症是剂量延迟反应的常见症状。
环磷酰胺+阿霉素+氟尿嘧啶	*XRCC1*	rs25487	CT 杂合型突变	药物毒副作用/有效性	Level 3	携带 CT 基因型的乳腺癌患者，相比于 CC 基因型，可能有较高发生恶心的风险。
环磷酰胺+阿霉素+氟尿嘧啶	*ERCC1*	rs3212986	AC 杂合型突变	药物毒副作用	Level 3	携带 AC 基因型的乳腺癌患者，相比于 AC 或 CC 基因型，可能有较低的嗜中性白血球减少症发生风险。
环磷酰胺+阿霉素+氟尿嘧啶	*ABCC4*	rs9561778	GT 杂合型突变	药物毒副作用	Level 3	携带 GT 基因型的乳腺癌患者，相比 GG 基因型，可能有较高的药物毒副风险。
环磷酰胺+阿霉素+氟尿嘧啶	*ATM*	rs1801516	GG 野生型	药物毒副作用	Level 3	携带 GG 基因型的乳腺癌患者，相比 AG 或 AA 基因型，可能有较低的恶性呕吐发生风险。
紫杉醇	*ABCB1*	rs1045642	AG 杂合型突变	药物毒副作用	Level 3	携带 AG 基因型的肿瘤患者，相比于 GG 基因型，可能有较高的嗜中性白血球减少症和神经毒性综合征。
紫杉醇	*ERCC1*	rs3212986	AC 杂合型突变	药物毒副作用	Level 3	携带 AC 基因型的乳腺癌患者，相比于 CC 基因型，可能有较高的周围神经病变风险。
紫杉醇	*SOD2*	rs4880	AG 杂合型突变	药物毒副作用	Level 3	携带 AG 基因型的乳腺癌患者，相比于 AA 基因型，可能有较低的周围神经病变风险，这种相关性只见于 *CYP3A4* 和 *CYP3A5* 基因功能缺失的患者。
紫杉醇	*EPHA5*	rs7349683	CT 杂合型突变	药物毒副作用	Level 3	携带 CT 基因型的乳腺癌或卵巢癌女性患者，相比 CC 基因型，可能有较高的外周神经病变发生风险；相比 TT 基因型，可能有较低的外周神经病变发生风险。然而，也有一项大型研究得出相反的结论。
多西他赛	*ERCC1*	rs11615	AG 杂合型突变	药物毒副作用	Level 3	携带 AG 基因型的乳腺癌患者，相比于 AA 基因型，可能有较低的黏膜炎风险，这种相关性只见于 *CYP3A4* 和 *CYP3A5* 基因功能缺失的患者。
多西他赛	*ERCC1*	rs3212986	AC 杂合型突变	药物毒副作用	Level 3	携带 AC 基因型的乳腺癌患者，相比于 CC 基因型，可能有较低的黏膜炎风险。
多西他赛	*ERCC2*	rs13181	TT 野生型	药物毒副作用	Level 3	携带 TT 基因型的乳腺癌患者，相比 GG 或 GT 基因型，可能有较高的嗜

报告的内容仅适用于专业的科学和医学研究人员进行使用和解读，不包含任何临床建议。
本报告内容可能涉及仍处于临床研究阶段的潜在药物或靶点，这种情况下会明确标出。
Copyright 北京泛生子基因科技有限公司 2020 Ref: Genetron P2003310111

						中性白血球减少症风险。
多西他赛+沙利度胺	*NAT2*	rs1799931	GG 野生型	药物毒副作用	Level 3	携带 GG 基因型的患者，相比 AA 基因型，可能有较高的毒性风险。
蒽环类	*ABCB1*	rs1045642	AG 杂合型突变	药物有效性	Level 3	携带 AG 基因型的乳腺癌患者，相比于 AA 基因型，可能有较低的药物完全缓解率。
甲氨喋呤	*MTHFR*	rs1801133	AA 纯合型突变	药物剂量/毒副作用/有效性	Level 2A	携带 AA 基因型的白血病或者淋巴瘤患者，相比于 AG 或 GG 基因型，可能有较差的药物缓解率，较高的药物毒副风险，较低的剂量和较高的叶酸缺乏症可能性。然而也存在不一致研究结果。
甲氨喋呤	*GGH*	rs11545078	GG 野生型	药物毒副作用	Level 3	携带 GG 基因型的肿瘤患者，相比于 AA 和 AG 基因型，可能有（1）较低的氨甲喋呤活性代谢物的蓄积；（2）较低的发生血小板减少症的风险。
甲氨喋呤	*GSTP1*	rs1695	AA 野生型	药物毒副作用	Level 3	携带 AA 基因型的前体淋巴母细胞淋巴瘤患者，相比于 GG 基因型，可能有较低的药物毒性风险。
甲氨喋呤	*MTHFR*	rs1801131	TT 野生型	药物有效性	Level 3	携带 TT 基因型的患者，相比于 GG 或 GT 基因型，有较高的无进展生存期；然而也有结论不一致性研究。
甲氨喋呤	*MTR*	rs1805087	AA 野生型	药物有效性/毒副作用	Level 3	携带 AA 基因型的骨肉瘤患者，相比于 GG 基因型，1）可能有较低药物毒副风险；2）可能有较高药物缓解率。
甲氨喋呤	*DHFR*	rs442767	TT 纯合型突变	药物毒副作用	Level 3	携带 TT 基因型的淋巴瘤患者，相比 GT 基因型，可能有较低的白细胞减少症风险。

注：1. 基因单核苷酸多态性与化疗药物相关性提示参考遗传药理学和基因组药理学 PharmGKB 数据库（http://www.pharmgkb.org），其证据等级划分依据如下：

Level 1A：由临床药理学实施联盟（CPIC）或遗传药理学指南认可；或者应用于国际遗传药理学研究网（PGRN）及其它主要卫生系统；

Level 1B：多项研究支持其与药物的相关性，且研究具有显著统计学差异；

Level 2A：多项研究支持其与药物的相关性，且该基因是已知的重要的功能明确的药物代谢基因；

Level 2B：多项研究支持其与药物的相关性，但其中一些研究无统计显著性或样本数量较少；

Level 3： 单一研究支持有显著差异或多项研究但尚未达成一致结果支持其相关性；

N/A：未划分证据等级，参考具有统计显著性的研究结论。

2. 化疗药物毒副作用和有效性也会受到其它一些遗传或临床因素的影响，存在个体差异性。

3. 杂合型突变提示化疗药物的有效性和毒副作用均为适中。

报告的内容仅适用于专业的科学和医学研究人员进行使用和解读，不包含任何临床建议。
本报告内容可能涉及仍处于临床研究阶段的潜在药物或靶点，这种情况下会明确标出。
Copyright 北京泛生子基因科技有限公司 2020 Ref: Genetron P2003310111

四、遗传性肿瘤风险提示

风险分析结果：未检测到与肿瘤遗传易感性相关的明确致病突变

注：1. 本次结果仅提示已检测基因的肿瘤遗传风险，并不能排除其它检测范围以外的致病突变的存在可能；且该部分的解读是基于目前已有的科学证据，随着科学技术的发展，可能会对已有知识和概念产生新的认识。

2. 结合受检者的家族患癌史，不排除以下两种情况仍会增加受检者的患癌风险：

（1）尚未有研究报告的 可能增加患癌风险的遗传性因素；

（2）家族共有的可能增加患癌风险的生活环境或方式。

报告的内容仅适用于专业的科学和医学研究人员进行使用和解读，不包含任何临床建议。
本报告内容可能涉及仍处于临床研究阶段的潜在药物或靶点，这种情况下会明确标出。
Copyright 北京泛生子基因科技有限公司 2020 Ref: Genetron P2003310111

五、检测结果详细解析

● 潜在获益靶向药物相关基因解析

TP53

基因变异	*TP53*-p.Arg283AlafsTer23
位点注释	*TP53* 基因编码的蛋白在第 283 位氨基酸开始发生了移码突变，此基因变异在样本中的变异频率为 92.7%。
位点解析	受检者样本携带的 *TP53*-p.Arg283AlafsTer23 突变是移码突变，尚未被 COSMIC 收录（COSMIC，Apr 2020）。移码突变会导致提前产生终止密码子，编码截短的蛋白产物，可能影响蛋白的功能。有研究表明，*TP53* 基因突变存在于 50%的脉络丛癌（CPC）患者中，与预后不良有关。并且 TP53 的缺失可能导致脉络丛乳头状瘤（CPP）向 CPC 的恶性进展 [PMID:30885981]。
基因描述	*TP53* 是一种肿瘤抑制基因，其突变或缺失会导致基因组不稳定和细胞过度增殖。*TP53* 基因编码肿瘤抑制蛋白，参与多种细胞应激、调节靶基因，诱导细胞周期停滞、细胞凋亡、衰老、DNA 修复和代谢改变。p53 蛋白在正常细胞中低表达，在恶性肿瘤中高表达。

潜在获益药物	MK-1775
FDA/NMPA 批准适应症	目前，FDA 尚未批准任何靶向 *TP53* 突变的药物。
NCCN 指南推荐	无
临床试验/回顾性分析	针对 *TP53* 突变的基因疗法、靶向肿瘤疫苗和抗癌药物正处于临床试验早期阶段。这些治疗方法包括 SGT-53、ALT-801、MK-1775 以及 Alisertib 等，它们在晚期实体肿瘤的治疗中表现出一定的疗效。一项 1 期临床试验结果显示，11 名难治性癌症患者接受 SGT-53 基因治疗后，7 名患者的病情稳定期达到了 6 周，中位生存期为 340 天 [PMID:23609015]。ALT-801 是一种靶向于 p53 蛋白的白细胞介素 2 融合物，ALT-801 的 1 期临床试验结果表明，10 例实体瘤患者达到了疾病稳定，维持时间为至少 11 周以上 [PMID:21994418]。Kevetrin 可以干扰 p53-MDM2 蛋白相互作用，并重新激活野生型 p53。MK-1775 是一种 Wee1 酪氨酸激酶抑制剂，Wee1 蛋白可以调控 G2 期细胞周期检查点。携带 *TP53* 突变的卵巢癌患者在接受 MK-1775 与化疗药物的联合治疗后，结果显示有较好的耐受性，无进展生存期延长 [ASCO 2015, Abstract 2507; ASCO 2015,

报告的内容仅适用于专业的科学和医学研究人员进行使用和解读，不包含任何临床建议。
本报告内容可能涉及仍处于临床研究阶段的潜在药物或靶点，这种情况下会明确标出。
Copyright 北京泛生子基因科技有限公司 2020 Ref: Genetron P2003310111

Abstract 5506]。另有研究表明，携带 *TP53* 突变的癌细胞对极光激酶 A 抑制剂敏感 [PMID:25200357]。一项评估 Alisertib 在实体瘤治疗中安全性和疗效的 2 期临床试验研究显示，药物客观药物缓解率（ORR）在乳腺癌患者、小细胞肺癌患者、非小细胞肺癌患者、头颈部鳞状细胞癌患者和胃腺癌患者依次分别为 18%（9/49）、21%（10/48）、4%（1/23）、9%（4/45）和 9%（4/47）。最常见的 3-4 级不良事件包括中性粒细胞减少症，白细胞减少症和贫血 [PMID:25728526]。此外，一项 1 期临床试验结果表明，极光激酶 A 抑制剂 AMG900 用于实体瘤患者，76.5% (13/17) 的患者达到疾病稳定，其中 1 例卵巢癌患者达到部分缓解，持续时间长达 6 个月，最常见的不良反应为中性粒细胞减少症 [ASCO 2009, Abstract 3009]。

个案报道	无
临床前期证据	无

潜在耐药药物	无
靶向耐药药物解析	无

潜在获益药物列表及获批、临床试验状态

药物通用名	商品名	靶点/原理	FDA/NMPA 批准于本癌种	FDA/NMPA 批准于其它癌种	2/3 期临床试验	1 期临床试验
MK-1775 暂无中文名	暂无	WEE1 酪氨酸激酶抑制剂			★	
Alisertib 暂无中文名	暂无	极光激酶 A 小分子激酶抑制剂				★
SGT-53 暂无中文名	暂无	*TP53* 基因治疗（通过靶向转铁蛋白的纳米颗粒进行药物传递）				★
Kevetrin 暂无中文名	暂无	干扰 P53-MDM2 蛋白相互作用，重新激活野生型 P53				★
ENMD-2076 暂无中文名	暂无	极光激酶 A 小分子激酶抑制剂				★
AT9283 暂无中文名	暂无	极光激酶 A，B/JAK2/JAK3/BCR-ABL 激酶抑制剂				★
AMG 900 暂无中文名	暂无	极光激酶 A,B,C/P38α/TYK2/JNK2/MET/TIE2 小分子激酶抑制剂				★
ALT-801	暂无	靶向 P53 蛋白的 T 细胞受体-				★

报告的内容仅适用于专业的科学和医学研究人员进行使用和解读，不包含任何临床建议。
本报告内容可能涉及仍处于临床研究阶段的潜在药物或靶点，这种情况下会明确标出。
Copyright 北京泛生子基因科技有限公司 2020 Ref: Genetron P2003310111

暂无中文名	白细胞介素 2 融合物

相关临床试验信息

靶向药物方案	适应症	临床试验招募状态	临床试验分期	临床试验地点	临床试验信息
MK-1775	中枢神经系统肿瘤	招募中	1/2 期	美国,加拿大	A Phase 1/2 Study of AZD1775 (MK-1775) in Combination With Oral Irinotecan in Children, Adolescents, and Young Adults With Relapsed or Refractory Solid Tumors https://ClinicalTrials.gov/show/NCT02095132
MK-1775	实体瘤	招募中	2 期	美国,波多黎各	NCI-MATCH: Targeted Therapy Directed by Genetic Testing in Treating Patients With Advanced Refractory Solid Tumors, Lymphomas, or Multiple Myeloma https://ClinicalTrials.gov/show/NCT02465060
Alisertib	脑肿瘤	已完成	1 期	美国	Alisertib and Fractionated Stereotactic Radiosurgery in Treating Patients With Recurrent High Grade Gliomas https://ClinicalTrials.gov/show/NCT02186509
Alisertib	实体瘤	已完成	1 期	美国	Study of MLN8237 in Participants With Advanced Solid Tumors https://ClinicalTrials.gov/show/NCT00962091
SGT-53	实体瘤	招募中	1 期	美国	A Study of SGT-53 in Children With Refractory or Recurrent Solid Tumors https://ClinicalTrials.gov/show/NCT02354547
SGT-53	实体瘤	已完成	1 期	美国	Safety Study of Infusion of SGT-53 to Treat Solid Tumors https://ClinicalTrials.gov/show/NCT00470613
Kevetrin	实体瘤	已完成	1 期	美国	A Safety, Pharmacokinetic and Pharmacodynamic Study of Kevetrin in Patients With Advanced Solid Tumors https://ClinicalTrials.gov/show/NCT01664000
ENMD-2076	实体瘤	已完成	1 期	美国	A Dose-Escalation Study of ENMD-2076 Administered Orally to Patients With Advanced Cancer https://ClinicalTrials.gov/show/NCT00658671
AT9283	实体瘤	已完成	1 期	加拿大	Aurora Kinase Inhibitor AT9283 in Treating Patients With Advanced or Metastatic Solid Tumors or Non-Hodgkin's Lymphoma https://ClinicalTrials.gov/show/NCT00443976
AT9283	实体瘤	已完成	1 期	英国	AT9283 in Children and Adolescents With

报告的内容仅适用于专业的科学和医学研究人员进行使用和解读，不包含任何临床建议。
本报告内容可能涉及仍处于临床研究阶段的潜在药物或靶点，这种情况下会明确标出。
Copyright 北京泛生子基因科技有限公司 2020 Ref: Genetron P2003310111

					Relapsed and Refractory Solid Tumors https://ClinicalTrials.gov/show/NCT00985868
AMG 900	实体瘤	已完成	1 期	美国,澳大利亚	A Phase 1 First-in-Human Study Evaluating AMG 900 in Advanced Solid Tumors https://ClinicalTrials.gov/show/NCT00858377
ALT-801	实体瘤	已完成	1 期	美国	Safety and Efficacy Study of ALT-801 to Treat Progressive Metastatic Malignancies https://ClinicalTrials.gov/show/NCT00496860

注：上述药物临床试验信息未经过临床试验准入条件进行筛选，上述临床试验信息仅供参考，更多临床试验准入条件等信息请以临床试验网站 https://clinicaltrials.gov/为准。

报告的内容仅适用于专业的科学和医学研究人员进行使用和解读，不包含任何临床建议。
本报告内容可能涉及仍处于临床研究阶段的潜在药物或靶点，这种情况下会明确标出。
Copyright 北京泛生子基因科技有限公司 2020 Ref: Genetron P2003310111

参考文献

1. Mutational burden of tumors with primary site unknown. ASCO 2017 Abstract.[ASCO 2017, Abstract 3039]
2. Estimating tumor mutation burden using next generation sequencing assay. ASCO 2017 Abstract.[ASCO 2017, Abstract e14529]
3. Carbone DP, Reck M, Paz-Ares L et al. First-Line Nivolumab in Stage IV or Recurrent Non-Small-Cell Lung Cancer. N Engl J Med 2017; 376: 2415-2426.[PMID:28636851]
4. Hause RJ, Pritchard CC, Shendure J, Salipante SJ. Classification and characterization of microsatellite instability across 18 cancer types. Nat Med 2016; 22: 1342-1350.[PMID:27694933]
5. Le DT, Uram JN, Wang H et al. PD-1 Blockade in Tumors with Mismatch-Repair Deficiency. N Engl J Med 2015; 372: 2509-2520.[PMID:26028255]
6. Hussein YR, Weigelt B, Levine DA et al. Clinicopathological analysis of endometrial carcinomas harboring somatic POLE exonuclease domain mutations. Mod Pathol 2015; 28: 505-514.[PMID:25394778]
7. van Gool IC, Eggink FA, Freeman-Mills L et al. POLE Proofreading Mutations Elicit an Antitumor Immune Response in Endometrial Cancer. Clin Cancer Res 2015; 21: 3347-3355.[PMID:25878334]
8. Bellone S, Centritto F, Black J et al. Polymerase epsilon (POLE) ultra-mutated tumors induce robust tumor-specific CD4+ T cell responses in endometrial cancer patients. Gynecol Oncol 2015; 138: 11-17.[PMID:25931171]
9. Association of POLE-mutated and MSI endometrial cancers with an elevated number of tumor-infiltrating and peritumoral lymphocytes and higher expression of PD-L1.[ASCO 2015, Abstract 5511]
10. Kandoth C, Schultz N, Cherniack AD et al. Integrated genomic characterization of endometrial carcinoma. Nature 2013; 497: 67-73.[PMID:23636398]
11. Mehnert JM, Panda A, Zhong H et al. Immune activation and response to pembrolizumab in POLE-mutant endometrial cancer. J Clin Invest 2016; 126: 2334-2340.[PMID:27159395]
12. Mittica G, Ghisoni E, Giannone G et al. Checkpoint inhibitors in endometrial cancer: preclinical rationale and clinical activity. Oncotarget 2017; 8: 90532-90544.[PMID:29163851]
13. Song J, Hong P, Liu C et al. Human POLD1 modulates cell cycle progression and DNA damage repair. BMC Biochem 2015; 16: 14.[PMID:26087769]
14. Tumini E, Barroso S, Calero CP, Aguilera A. Roles of human POLD1 and POLD3 in genome stability. Sci Rep 2016; 6: 38873.[PMID:27974823]
15. Palles C, Cazier JB, Howarth KM et al. Germline mutations affecting the proofreading domains of POLE and POLD1 predispose to colorectal adenomas and carcinomas. Nat Genet 2013; 45: 136-144.[PMID:23263490]
16. Kato S, Goodman A, Walavalkar V et al. Hyperprogressors after Immunotherapy: Analysis of Genomic Alterations Associated with Accelerated Growth Rate. Clin Cancer Res 2017; 23: 4242-4250.[PMID:28351930]
17. 1140PDPredictive biomarkers for hyper-progression (HP) in response to immune checkpoint inhibitors (ICI)–analysis of somatic alterations (SAs).[EMSO 2017,1140PD]
18. Ju W, Chen S, Wang G, et al. Association between MDM2/MDM4 amplification and PD-1/PD-L1 inhibitors-related hyperprogressive disease: A pan-cancer analysis. 2019.[ASCO 2019, Abstract 2557]
19. Singavi A K, Menon S, Kilari D, et al. 1140PDPredictive biomarkers for hyper-progression (HP) in response to immune checkpoint inhibitors (ICI)–analysis of somatic alterations (SAs). Annals of Oncology, 2017, 28(suppl_5).[Annals of Oncology, 2017, 28(suppl_5)]
20. Sade-Feldman M, Jiao Y J, Chen J H, et al. Resistance to checkpoint blockade therapy through inactivation of

报告的内容仅适用于专业的科学和医学研究人员进行使用和解读，不包含任何临床建议。
本报告内容可能涉及仍处于临床研究阶段的潜在药物或靶点，这种情况下会明确标出。
Copyright 北京泛生子基因科技有限公司 2020 Ref: Genetron P2003310111

antigen presentation. Nature communications, 2017, 8(1): 1136.[PMID:29070816]

21. Yan X, Zhang S, Deng Y, et al. Prognostic Factors for Checkpoint Inhibitor Based Immunotherapy: An Update With New Evidences. Frontiers in pharmacology, 2018, 9.[PMID:30294272]
22. Zhao J, Chen A X, Gartrell R D, et al. Immune and genomic correlates of response to anti-PD-1 immunotherapy in glioblastoma. Nature medicine, 2019, 25(3): 462.[PMID:30742119]
23. Herbst RS, Baas P, Kim DW et al. Pembrolizumab versus docetaxel for previously treated, PD-L1-positive, advanced non-small-cell lung cancer (KEYNOTE-010): a randomised controlled trial. Lancet 2016; 387: 1540-1550.[PMID:26712084]
24. Borghaei H, Paz-Ares L, Horn L et al. Nivolumab versus Docetaxel in Advanced Nonsquamous Non-Small-Cell Lung Cancer. N Engl J Med 2015; 373: 1627-1639.[PMID:26412456]
25. Fehrenbacher L, Spira A, Ballinger M et al. Atezolizumab versus docetaxel for patients with previously treated non-small-cell lung cancer (POPLAR): a multicentre, open-label, phase 2 randomised controlled trial. Lancet 2016; 387: 1837-1846.[PMID:26970723]
26. Lee CK, Man J, Lord S et al. Checkpoint Inhibitors in Metastatic EGFR-Mutated Non-Small Cell Lung Cancer-A Meta-Analysis. J Thorac Oncol 2017; 12: 403-407.[PMID:27765535]
27. Peters S, Gettinger S, Johnson ML et al. Phase II Trial of Atezolizumab As First-Line or Subsequent Therapy for Patients With Programmed Death-Ligand 1-Selected Advanced Non-Small-Cell Lung Cancer (BIRCH). J Clin Oncol 2017; 35: 2781-2789.[PMID:28609226]
28. Brahmer J, Reckamp KL, Baas P et al. Nivolumab versus Docetaxel in Advanced Squamous-Cell Non-Small-Cell Lung Cancer. N Engl J Med 2015; 373: 123-135.[PMID:26028407]
29. Gainor JF, Shaw AT, Sequist LV et al. EGFR Mutations and ALK Rearrangements Are Associated with Low Response Rates to PD-1 Pathway Blockade in Non-Small Cell Lung Cancer: A Retrospective Analysis. Clin Cancer Res 2016; 22: 4585-4593.[PMID:27225694]
30. Sabari J K, Leonardi G C, Shu C A, et al. PD-L1 expression, tumor mutational burden, and response to immunotherapy in patients with MET exon 14 altered lung cancers. Annals of Oncology, 2018, 29(10): 2085-2091.[PMID:30165371]
31. Baba K, Tanaka H, Sakamoto H, et al. Efficacy of pembrolizumab for patients with both high PD-L1 expression and an MET exon 14 skipping mutation: A case report. Thoracic cancer, 2019, 10(2): 369-372.[PMID:30600919]
32. Dong ZY, Zhong WZ, Zhang XC et al. Potential Predictive Value of TP53 and KRAS Mutation Status for Response to PD-1 Blockade Immunotherapy in Lung Adenocarcinoma. Clin Cancer Res 2017; 23: 3012-3024.[PMID:28039262]
33. Serra P, Petat A, Maury JM et al. Programmed cell death-ligand 1 (PD-L1) expression is associated with RAS/TP53 mutations in lung adenocarcinoma. Lung Cancer 2018; 118: 62-68.[PMID:29572005]
34. Meraz IM, Majidi M, Cao X et al. TUSC2 Immunogene Therapy Synergizes with Anti-PD-1 through Enhanced Proliferation and Infiltration of Natural Killer Cells in Syngeneic Kras-Mutant Mouse Lung Cancer Models. Cancer Immunol Res 2018; 6: 163-177.[PMID:29339375]
35. Lan B, Ma C, Zhang C et al. Association between PD-L1 expression and driver gene status in non-small-cell lung cancer: a meta-analysis. Oncotarget 2018; 9: 7684-7699.[PMID:29484144]
36. Koyama S, Akbay EA, Li YY et al. STK11/LKB1 Deficiency Promotes Neutrophil Recruitment and Proinflammatory Cytokine Production to Suppress T-cell Activity in the Lung Tumor Microenvironment. Cancer Res 2016; 76: 999-1008.[PMID:26833127]
37. Shackelford DB, Shaw RJ. The LKB1-AMPK pathway: metabolism and growth control in tumour suppression. Nat Rev Cancer 2009; 9: 563-575.[PMID:19629071]

报告的内容仅适用于专业的科学和医学研究人员进行使用和解读，不包含任何临床建议。
本报告内容可能涉及仍处于临床研究阶段的潜在药物或靶点，这种情况下会明确标出。
Copyright 北京泛生子基因科技有限公司 2020 Ref: Genetron P2003310111

38. Skoulidis F, Goldberg ME, Greenawalt DM et al. STK11/LKB1 Mutations and PD-1 Inhibitor Resistance in KRAS-Mutant Lung Adenocarcinoma. Cancer Discov 2018; 8: 822-835.[PMID:29773717]
39. Ivashkiv LB. IFNgamma: signalling, epigenetics and roles in immunity, metabolism, disease and cancer immunotherapy. Nat Rev Immunol 2018; 18: 545-558.[PMID:29921905]
40. Shin DS, Zaretsky JM, Escuin-Ordinas H et al. Primary Resistance to PD-1 Blockade Mediated by JAK1/2 Mutations. Cancer Discov 2017; 7: 188-201.[PMID:27903500]
41. Association of CDKN2A gene alteration with high expression of PD-L1.[ASCO 2018,Abstract 9102]
42. Assessment of treatment response to immunotherapy in melanoma patients with pathogenic mutations of NRAS, BRAF, CDKN2A and P53.[ASCO 2017,Abstract e21057]
43. Horn S, Leonardelli S, Sucker A et al. Tumor CDKN2A-Associated JAK2 Loss and Susceptibility to Immunotherapy Resistance. J Natl Cancer Inst 2018; 110: 677-681.[PMID:29917141]
44. TUMOUR CDKN2A LOSS PREDISPOSES TO IMMUNOTHERAPY RESISTANCE.[ESMO 2018,PO-399]
45. Peng W, Chen JQ, Liu C et al. Loss of PTEN Promotes Resistance to T Cell-Mediated Immunotherapy. Cancer Discov 2016; 6: 202-216.[PMID:26645196]
46. Johnson DB, Lovly CM, Flavin M et al. Impact of NRAS mutations for patients with advanced melanoma treated with immune therapies. Cancer Immunol Res 2015; 3: 288-295.[PMID:25736262]
47. Recurrent SERPINB3 and SERPINB4 mutations in patients who respond to anti-CTLA4 immunotherapy.[PMID:27668655]
48. Riaz N, Havel J J, Makarov V, et al. Tumor and microenvironment evolution during immunotherapy with nivolumab. Cell, 2017, 171(4): 934-949. e16.[PMID:29033130]
49. Patel SJ, Sanjana NE, Kishton RJ et al. Identification of essential genes for cancer immunotherapy. Nature 2017; 548: 537-542.[PMID:28783722]
50. Ni L, Lu J. Interferon gamma in cancer immunotherapy. Cancer Med 2018; 7: 4509-4516.[PMID:30039553]
51. Gao J, Shi L Z, Zhao H, et al. Loss of IFN-r pathway genes in tumor cells as a mechanism of resistance to anti-CTLA-4 therapy. Cell, 2016, 167(2): 397-404. e9.[PMID:27667683]
52. Manguso R T, Pope H W, Zimmer M D, et al. In vivo CRISPR screening identifies Ptpn2 as a cancer immunotherapy target. Nature, 2017, 547(7664): 413.[PMID:28723893]
53. Spranger S, Bao R, Gajewski T F. Melanoma-intrinsic B-catenin signalling prevents anti-tumour immunity. Nature, 2015, 523(7559): 231.[PMID:25970248]
54. Miao D, Margolis C A, Gao W, et al. Genomic correlates of response to immune checkpoint therapies in clear cell renal cell carcinoma. Science, 2018, 359(6377): 801-806.[PMID:29301960]
55. Wang J, Merino DM, Light N, et al. Myc and Loss of p53 Cooperate to Drive Formation of Choroid Plexus Carcinoma. Cancer Res. 2019;79(9):2208–2219. [PMID:30885981]
56. Senzer N, Nemunaitis J, Nemunaitis D et al. Phase I study of a systemically delivered p53 nanoparticle in advanced solid tumors. Mol Ther 2013; 21: 1096-1103.[PMID:23609015]
57. Fishman MN, Thompson JA, Pennock GK et al. Phase I trial of ALT-801, an interleukin-2/T-cell receptor fusion protein targeting p53 (aa264-272)/HLA-A*0201 complex, in patients with advanced malignancies. Clin Cancer Res 2011; 17: 7765-7775.[PMID:21994418]
58. Leijen S vGR, Sonke G S, et al. Phase II study with Wee1 inhibitor AZD1775 plus carboplatin in patients with p53 mutated ovarian cancer refractory or resistant (<3 months) to standard first line therapy. Journal of Clinical Oncology 2015; 2507-2507.[ASCO 2015, Abstract 2507]
59. Amit M. Oza JIW, Diane M. Provencher et al. An international, biomarker-directed, randomized, phase II trial of AZD1775 plus paclitaxel and carboplatin (P/C) for the treatment of women with platinum-sensitive, TP53-mutant

报告的内容仅适用于专业的科学和医学研究人员进行使用和解读，不包含任何临床建议。
本报告内容可能涉及仍处于临床研究阶段的潜在药物或靶点，这种情况下会明确标出。
Copyright 北京泛生子基因科技有限公司 2020 Ref: Genetron P2003310111

ovarian cancer.[ASCO 2015, Abstract 5506]

60. Malumbres M, Perez de Castro I. Aurora kinase A inhibitors: promising agents in antitumoral therapy. Expert Opin Ther Targets 2014; 18: 1377-1393.[PMID:25200357]

61. Melichar B, Adenis A, Lockhart AC et al. Safety and activity of alisertib, an investigational aurora kinase A inhibitor, in patients with breast cancer, small-cell lung cancer, non-small-cell lung cancer, head and neck squamous-cell carcinoma, and gastro-oesophageal adenocarcinoma: a five-arm phase 2 study. Lancet Oncol 2015; 16: 395-405.[PMID:25728526]

62. Carducci MA, Shaheen MF, Paller CJ et al. First-in-human study of AMG 900, an oral pan-Aurora kinase inhibitor, in adult patients (pts) with advanced solid tumors.[ASCO 2009, Abstract 3009]

报告的内容仅适用于专业的科学和医学研究人员进行使用和解读，不包含任何临床建议。
本报告内容可能涉及仍处于临床研究阶段的潜在药物或靶点，这种情况下会明确标出。
Copyright 北京泛生子基因科技有限公司 2020 Ref: Genetron P2003310111

第三部分：附录

一、慈善援助药物信息

药物名称	纳武利尤单抗（欧狄沃）
FDA 批准适应症	FDA 批准纳武利尤单抗（Nivolumab）用于非小细胞肺癌、黑色素瘤、肾细胞癌、经典型霍奇金淋巴瘤、头颈部鳞状细胞癌、尿路上皮癌、结直肠癌和肝细胞癌的治疗。<br>NMPA 批准纳武利尤单抗（Nivolumab）用于 *EGFR* 基因突变阴性和 *ALK* 阴性、既往接受过含铂方案化疗后疾病进展或不可耐受的局部晚期或转移性 NSCLC 患者的治疗。
赞助公司	百时美施贵宝公司
医学标准	单药适用于表皮生长因子受体（*EGFR*）基因突变阴性和间变性淋巴瘤激酶（ALK）阴性、既往接受过含铂方案化疗后疾病进展或不可耐受的局部晚期或转移性非小细胞肺癌（NSCLC）的成人患者。<br>经指定医疗机构评估，确诊为适用于单药治疗接受含铂类方案治疗期间或之后出现疾病进展且肿瘤 PD-L1 表达阳性 （定义为表达 PD-L1 的肿瘤细胞≥1%）的复发性或转移性头颈部鳞状细胞癌（SCCHN)患者。
官网链接	http://www.cfchina.org.cn/list.php?catid=439

药物名称	度伐利尤单抗(英飞凡)
FDA 批准适应症	FDA 批准度伐利尤单抗(Durvalumab)用于尿路上皮癌和非小细胞肺癌的治疗。<br>NMPA 批准度伐利尤单抗（Durvalumab）用于治疗同步放化疗后未进展的不可切除的 III 期非小细胞肺癌。
赞助公司	阿斯利康投资（中国）有限公司
医学标准	1.经病理学或细胞学证实符合英飞凡适应症：在接受铂类药物为基础的化疗同步放疗后未

报告的内容仅适用于专业的科学和医学研究人员进行使用和解读，不包含任何临床建议。
本报告内容可能涉及仍处于临床研究阶段的潜在药物或靶点，这种情况下会明确标出。
Copyright 北京泛生子基因科技有限公司 2020 Ref: Genetron P2003310111

	出现疾病进展的不可切除、III 期非小细胞肺癌（NSCLC）;<br>2.在度伐利尤注射液（英飞凡）治疗之前必须接受铂类药物为基础的化疗同步放疗后未出现疾病进展；<br>3.接受度伐利尤注射液（英飞凡）治疗期间不得同时进行其他 PD-1 或 PD-L1 抑制剂和化疗药物的治疗；<br>4.患者身体条件可以耐受肿瘤免疫治疗；<br>5.有足够的临床证据证实患者能够从英飞凡治疗中获益且无严重不良反应（获益是指肿瘤病灶按照 RECIST 1.1 标准没有肿瘤进展；无严重不良反应是指未发生因度伐利尤注射液（英飞凡）治疗引起的不可逆转或者不可耐受的伤害）。
官网链接	https://www.ilvzhou.com/index.php?m=content&c=index&a=lists&catid=189

药物名称	哌柏西利（爱博新）
FDA 批准适应症	FDA 批准 CDK4/6 抑制剂哌柏西利用于乳腺癌的治疗。<br>NMPA 批准哌柏西利用于乳腺癌的治疗。
赞助公司	辉瑞公司
医学标准	经医学评估确认为符合哌柏西利胶囊（爱博新）在中国获批适应症。经过哌柏西利胶囊（爱博新）持续治疗获得明确疗效且无不可耐受不良反应，且经济上无法支付持续服用哌柏西利胶囊（爱博新）治疗费用中国大陆患者。
官网链接	http://baxs.ilvzhou.com/

药物名称	帕博利珠单抗（可瑞达）
FDA 批准适应症	FDA 批准帕博利珠单抗用于晚期、不可切除的或转移性黑色素瘤、非小细胞肺癌、头颈癌、高微卫星不稳定性癌症、胃癌、宫颈癌、原发性纵膈大 B 细胞淋巴瘤、尿路上皮癌、结直肠癌、弥漫性大 B 细胞淋巴瘤、肝癌、头颈部鳞状细胞癌、泌尿生殖系统癌症、柱状癌（Merkel 细胞癌）和小细胞肺癌的治疗。<br>NMPA 批准帕博利珠单抗用于一线治疗失败的不可切除或转移性黑色素瘤的治疗。
赞助公司	默沙东（中国）有限公司
医学标准	符合帕博利珠单抗注射液（可瑞达）中国适应症的患者：<br>1. PD-L1 阳性晚期非小细胞肺癌一线单药治疗适应症：<br>1）经病理学（组织学或细胞学）确诊的鳞状或非鳞状非小细胞肺癌（NSCLC ）。<br>2）临床分期为局部晚期或转移性疾病。

报告的内容仅适用于专业的科学和医学研究人员进行使用和解读，不包含任何临床建议。
本报告内容可能涉及仍处于临床研究阶段的潜在药物或靶点，这种情况下会明确标出。
Copyright 北京泛生子基因科技有限公司 2020 Ref: Genetron P2003310111

	3）既往未接受过针对局部晚期或转移性非小细胞肺癌的全身性抗肿瘤治疗。<br>4）患者身体条件可以耐受肿瘤免疫治疗。<br>5）排除表皮生长因子受体（*EGFR*）敏感基因突变阳性和间变性淋巴瘤激酶（ALK）阳性的病人。<br>6）针对最初单药治疗的患者必须做 PD-L1 检测，且 PD-L1 TPS≥1%。<br>2. 晚期非鳞状非小细胞肺癌一线联合化疗治疗适应症：<br>1）经病理学（组织学或细胞学）确诊的非鳞状非小细胞肺癌（NSCLC）。<br>2）临床分期为转移性疾病。<br>3）既往未接受过针对转移性非小细胞肺癌的全身性抗肿瘤治疗。<br>4）患者身体条件可以耐受肿瘤免疫治疗和化学治疗。<br>5）排除表皮生长因子受体（*EGFR*）敏感基因突变阳性和间变性淋巴瘤激酶（ALK）阳性的病人。<br>3. 晚期鳞状非小细胞肺癌一线联合化疗治疗适应症<br>1）经病理学（组织学或细胞学）确诊的鳞状非小细胞肺癌(NSCLC)。<br>2）临床分期为转移性疾病。<br>3）既往未接受过针对转移性非小细胞肺癌的全身性抗肿瘤治疗。<br>4）患者身体条件可以耐受肿瘤免疫治疗和化学治疗。<br>4. 二线黑色素瘤适应症：<br>1）经病理学（组织学或细胞学）确诊的 IV 期黑色素瘤患者，或不能接受根治性治疗的 III 期黑色素瘤患者。<br>2）必须是接受过一线治疗的晚期黑色素瘤患者。<br>3）患者一般情况适合肿瘤免疫治疗。
官网链接	http://smzy.ilvzhou.com

药物名称	仑伐替尼(乐卫玛)
FDA 批准适应症	FDA 批准仑伐替尼用于局部复发或进展性及放射性碘-难治性分化型甲状腺癌患者的治疗，与 mTOR 抑制剂依维莫司联用治疗用于既往接受抗血管生成治疗的晚期肾细胞癌（RCC）的治疗，及无法切除的肝细胞癌(HCC)患者的一线治疗；此外，FDA 还批准仑伐替尼联合帕博利珠单抗（可瑞达）组合突破性疗法用于不能局部治疗的晚期不可切除的肝细胞癌（HCC）患者的一线疗法。<br>NMPA 批准酪氨酸激酶抑制剂乐卫玛（甲磺酸仑伐替尼）单药在中国用于治疗既往未接受过全身系统治疗的不可切除的肝癌患者。
赞助公司	卫材公司
医学标准	1.项目医生评估申请人的疾病符合国家食品药品监督管理总局批准的仑伐替尼适应症。<br>2.由项目医院的项目医生评估确认患者符合入组医学标准。<br>3.患者需定期亲自到项目医生处复诊，确认符合继续使用的医学条件。
官网链接	http://waxh.huanzheyuanzhu.cn/

报告的内容仅适用于专业的科学和医学研究人员进行使用和解读，不包含任何临床建议。
本报告内容可能涉及仍处于临床研究阶段的潜在药物或靶点，这种情况下会明确标出。
Copyright 北京泛生子基因科技有限公司 2020 Ref: Genetron P2003310111

药物名称	特瑞普利单抗注射液（拓益）
FDA 批准适应症	NMPA 批准特瑞普利单抗治疗既往接受全身系统治疗失败的不可切除或转移性黑色素瘤
赞助公司	上海君实生物医药科技股份有限公司
医学标准	患者本人是经项目医生确诊需要特瑞普利单抗注射液（拓益）治疗的恶性黑色素瘤患者。患者本人需要连续使用一段时间特瑞普利单抗注射液（拓益）治疗后，依据项目医生临床评估，需要继续使用特瑞普利单抗注射液（拓益）治疗，并从中获益且无不可耐受的毒副反应。<br>患者本人需根据项目流程规定的时间接受医学随访，并提供相应的检查报告（包括：门诊或住院病历、处方单以及相关的医学检验报告），由项目医院提供评估报告，确认需继续使用特瑞普利单抗注射液（拓益）治疗。
官网链接	http://www.bqety.com/static/tuoyi/index.html

二、已纳入医保的肿瘤药物相关信息

药物名称	阿来替尼
FDA 批准适应症	目前，FDA 已批准阿来替尼（Alectinib）用于 ALK 阳性的转移性 NSCLC 患者的治疗。
剂型	口服常释剂型
医保支付标准	暂无
限定支付范围	限间变性淋巴瘤激酶（ALK）阳性的局部晚期或转移性非小细胞肺癌患者。

药物名称	阿昔替尼
FDA 批准适应症	FDA 已批准阿昔替尼（Axitinib）用于接受系统性治疗后失败的晚期肾细胞癌患者的治疗。

报告的内容仅适用于专业的科学和医学研究人员进行使用和解读，不包含任何临床建议。
本报告内容可能涉及仍处于临床研究阶段的潜在药物或靶点，这种情况下会明确标出。
Copyright 北京泛生子基因科技有限公司 2020 Ref: Genetron P2003310111

剂型	口服常释剂型
医保支付标准	207 元（5mg/片);<br>60.4 元（1mg/片）
限定支付范围	限既往接受过一种酪氨酸激酶抑制剂或细胞因子治疗失败的进展期肾细胞癌(RCC)的成人患者。

药物名称	贝伐珠单抗
FDA 批准适应症	FDA 已批准 Vegf 单克隆抗体贝伐珠单抗（Bevacizumab）用于转移性结直肠癌、胶质母细胞瘤、非小细胞肺癌、肾细胞癌、宫颈癌、复发性卵巢上皮癌、输卵管或原发性腹膜癌的治疗。
剂型	注射剂
医保支付标准	1998 元（100mg（4ml）/瓶）
限定支付范围	限晚期转移性结直肠癌或晚期非鳞非小细胞肺癌。

药物名称	克唑替尼
FDA 批准适应症	FDA 已批准多靶点激酶抑制剂克唑替尼（Crizotinib）用于 ALK 阳性或 ROS1 阳性的转移性非小细胞肺癌患者的治疗。
剂型	口服常释剂型
医保支付标准	260 元（250mg/粒);<br>219.2 元（200mg/粒）
限定支付范围	限间变性淋巴瘤激酶（ALK）阳性的局部晚期或转移性非小细胞肺癌患者或 ROS1 阳性的晚期非小细胞肺癌患者。

药物名称	尼妥珠单抗
FDA 批准适应症	（NMPA 批准适应症）试用于与放疗联合治疗表皮生长因子受体（EGFR）表达阳性的

报告的内容仅适用于专业的科学和医学研究人员进行使用和解读，不包含任何临床建议。
本报告内容可能涉及仍处于临床研究阶段的潜在药物或靶点，这种情况下会明确标出。
Copyright 北京泛生子基因科技有限公司 2020 Ref: Genetron P2003310111

	III/IV 期鼻咽癌。
剂型	注射剂
医保支付标准	1700 元（10ml：50mg/瓶）
限定支付范围	限与放疗联合治疗表皮生长因子受体(EGFR)表达阳性的 III/IV 期鼻咽癌。

药物名称	曲妥珠单抗
FDA 批准适应症	FDA 已批准曲妥珠单抗（Trastuzumab）用于 Her2 阳性的转移性胃腺癌或胃食管结合部腺癌患者的治疗；FDA 批准抗曲妥珠单抗用于 HER2 过表达的乳腺癌患者；FDA 批准帕妥珠单抗（Pertuzumab）联合曲妥珠单抗和多西他赛用于 HER2 阳性的、既往未曾接受抗-HER2 治疗或化疗的转移性乳腺癌患者。
剂型	注射剂
医保支付标准	7600 元（440mg（20ml）/瓶）
限定支付范围	限以下情况方可支付：<br>1.HER2 阳性的乳腺癌手术后患者，支付不超过 12 个月。<br>2.HER2 阳性的转移性乳腺癌。<br>3.HER2 阳性的晚期转移性胃癌。

药物名称	帕妥珠单抗
FDA 批准适应症	FDA 已批准抗 HER2 单克隆抗体帕妥珠单抗（Pertuzumab）联合曲妥珠单抗和多西他赛用于 HER2 阳性的、既往未曾接受抗-HER2 治疗或化疗的转移性乳腺癌患者
剂型	注射剂
医保支付标准	暂无
限定支付范围	限以下情况方可支付，且支付不超过 12 个月：<br>1.HER2 阳性的局部晚期、炎性或早期乳腺癌患者的新辅助治疗。<br>2.具有高复发风险 HER2 阳性早期乳腺癌患者的辅助治疗。

报告的内容仅适用于专业的科学和医学研究人员进行使用和解读，不包含任何临床建议。
本报告内容可能涉及仍处于临床研究阶段的潜在药物或靶点，这种情况下会明确标出。
Copyright 北京泛生子基因科技有限公司 2020 Ref: Genetron P2003310111

药物名称	信迪利单抗
FDA 批准适应症	（NMPA 批准适应症）批准至少经过二线系统化疗的复发或难治性经典型霍奇金淋巴瘤的治疗
剂型	注射剂
医保支付标准	2843 元（10ml:100mg/瓶）
限定支付范围	限至少经过二线系统化疗的复发或难治性经典型霍奇金淋巴瘤的患者。

药物名称	舒尼替尼
FDA 批准适应症	FDA 已批准舒尼替尼（Sunitinib）用于胃肠道间质瘤、晚期肾细胞癌及胰腺内分泌肿瘤的治疗。
剂型	口服常释剂型
医保支付标准	448 元（50mg/粒);<br>359.4 元（37.5mg/粒);<br>263.5 元（25mg/粒);<br>155 元（12.5mg/粒)
限定支付范围	1.不能手术的晚期肾细胞癌（RCC)；<br>2.甲磺酸伊马替尼治疗失败或不能耐受的胃肠间质瘤（GIST)；<br>3.不可切除的，转移性高分化进展期胰腺神经内分泌瘤（pNET）成人患者。

药物名称	西妥昔单抗
FDA 批准适应症	FDA 已批准 Egfr 单克隆抗体西妥昔单抗（Cetuximab）用于 KRAS 野生型结直肠癌的治疗与用于头颈鳞状细胞癌的治疗。
剂型	注射剂
医保支付标准	1295 元（100mg(20ml)/瓶）
限定支付范围	限 RAS 基因野生型的转移性结直肠癌。

报告的内容仅适用于专业的科学和医学研究人员进行使用和解读，不包含任何临床建议。
本报告内容可能涉及仍处于临床研究阶段的潜在药物或靶点，这种情况下会明确标出。
Copyright 北京泛生子基因科技有限公司 2020 Ref: Genetron P2003310111

药物名称	依维莫司
FDA 批准适应症	FDA 批准依维莫司（Everolimus）用于：<br>（1）与依西美坦联合治疗，用于来曲唑或阿那曲唑治疗失败后的，激素受体阳性、Her-2 阴性晚期乳腺癌适用激素治疗的患有乳腺癌的绝经后妇女。<br>（2）不可切除的、局部晚期或转移性的、分化良好的（中度分化或高度分化）进展期胰腺神经内分泌瘤成人患者。<br>（3）既往接受舒尼替尼或索拉非尼治疗失败的晚期肾细胞癌成人患者。<br>（4）不需要立即手术切除的成人结节性硬化症（TSC）和肾血管肌脂肪瘤。<br>（5）需要治疗干预但不适于手术切除的结节性硬化症相关的室管膜下巨细胞星形细胞瘤（SEGA）成人和 1 岁以上儿童患者。<br>（6）作为辅助治疗药物，用于与结节性硬化症相关的部分性癫痫成人和 2 岁以上儿童患者。
剂型	口服常释剂型
医保支付标准	148 元（5mg/片）<br>87.05 元（2.5mg/片）
限定支付范围	限以下情况方可支付：<br>1.接受舒尼替尼或索拉非尼治疗失败的晚期肾细胞癌成人患者。<br>2.不 可切除的、局部晚期或转移性的、分化良好的（中度分化或高度分化）进展期胰腺神经内分泌瘤成人患者。<br>3.无法手术切除的、局部晚期 或转移性的、分化良好的、进展期非功能性胃肠道或肺源神经内分泌肿瘤患者。<br>4.不需立即手术治疗的结节性硬化症相关的肾血管平滑肌脂肪瘤（TSC-AML)成人患者。<br>5.不能手术的结节性硬化症相关的室管膜下巨细胞星型细胞瘤的患者。

药物名称	阿法替尼
FDA 批准适应症	FDA 已批准阿法替尼（Afatinib）用于携带 EGFR 19 号外显子缺失或者 21 号外显子 Leu858Arg 突变的转移性 NSCLC 患者的一线治疗；用于铂类为基础的一线化疗后疾病进展的晚期肺鳞状细胞癌患者的治疗。
剂型	口服常释剂型
医保支付标准	200 元（40mg/片);<br>160.5 元（30mg/片）

报告的内容仅适用于专业的科学和医学研究人员进行使用和解读，不包含任何临床建议。
本报告内容可能涉及仍处于临床研究阶段的潜在药物或靶点，这种情况下会明确标出。
Copyright 北京泛生子基因科技有限公司 2020 Ref: Genetron P2003310111

限定支付范围	1.具有 *EGFR* 基因敏感突变的局部晚期或转移性非小细胞肺癌，既往未接受过 EGFR-TKI 治疗。<br>2.含铂化疗期间或化疗后疾病进展的局部晚期或转移性鳞状组织学类型的非小细胞肺癌。

药物名称	阿帕替尼
FDA 批准适应症	（NMPA 批准适应症）适用于既往至少接受过 2 种系统化疗后进展或复发的晚期胃腺癌或胃-食管结合部腺癌患者。
剂型	口服常释剂型
医保支付标准	115 元（250mg/片）<br>156.86 元（375mg/片）<br>172.63 元（425mg/片）
限定支付范围	限既往至少接受过 2 种系统化疗后进展或复发的晚期胃腺癌或胃-食管结合部腺癌患者。

药物名称	安罗替尼
FDA 批准适应症	（NMPA 批准适应症）适用于既往至少接受过 2 种系统化疗后出现进展或复发的局部晚期或转移性非小细胞肺癌患者的治疗。对于存在表皮生长因子（*EGFR*）基因突变或间变性淋巴瘤激酶（ALK）阳性的患者，在开始本品治疗前应接受相应的靶向药物治疗后进展，且至少接受过 2 中系统化疗后出现进展或复发。
剂型	口服常释剂型
医保支付标准	487 元（12mg/粒);<br>423.6 元（10mg/粒);<br>357 元（8mg/粒)
限定支付范围	限既往至少接受过 2 种系统化疗后出现进展或复发的局部晚期或转移性非小细胞肺癌患者。

药物名称	奥希替尼
FDA 批准适应症	FDA 已批准奥希替尼（Osimertinib）用于 Egfr 酪氨酸激酶抑制剂治疗后疾病进展的、

报告的内容仅适用于专业的科学和医学研究人员进行使用和解读，不包含任何临床建议。
本报告内容可能涉及仍处于临床研究阶段的潜在药物或靶点，这种情况下会明确标出。
Copyright 北京泛生子基因科技有限公司 2020 Ref: Genetron P2003310111

	携带 EGFR-Thr790Met 突变的晚期非小细胞肺癌患者的治疗。
剂型	口服常释剂型
医保支付标准	510 元（80mg/片);<br>300 元（40mg/片）
限定支付范围	限既往因表皮生长因子受体（EGFR）酪氨酸激酶抑制剂（TKI）治疗时或治疗后出现疾病进展，并且经检验确认存在 *EGFR* T790M 突变阳性的局部晚期或转移性非小细胞肺癌成人患者。

药物名称	奥拉帕利
FDA 批准适应症	FDA 已批准 PARP 抑制剂奥拉帕利（Olaparib）用于对一线铂类化疗敏感的、携带 *BRCA1/BRCA2* 有害突变或疑似有害突变(胚系突变/体细胞突变)的晚期上皮性卵巢癌、输卵管癌或原发性腹膜癌患者；批准用于对铂类化疗敏感的成人复发性上皮性卵巢癌、输卵管癌或原发性腹膜癌患者；批准用于接受过三种或三种以上化疗方案的、携带 *BRCA1/BRCA2* 有害突变或疑似有害突变（胚系突变）的晚期卵巢癌患者的治疗；还被批准用于治疗携带 *BRCA1/BRCA2* 有害突变或疑似有害突变（胚系突变）、HER2 阴性、曾接受过化疗的转移性乳腺癌患者。
剂型	口服常释剂型
医保支付标准	暂无
限定支付范围	限铂敏感的复发性上皮性卵巢癌、输卵管癌或原发性腹膜癌患者。

药物名称	吡咯替尼
FDA 批准适应症	（NMPA 批准适应症）适用于 EGFR/HER2 双靶点小分子激酶抑制剂吡咯替尼联合卡培他滨用于治疗 HER2 阳性、既往未接受或接受过曲妥珠单抗的复发或转移性乳腺癌患者
剂型	口服常释剂型
医保支付标准	暂无
限定支付范围	限表皮生长因子受体 2（HER2）阳性的复发或转移性乳腺癌患者的二线治疗。

报告的内容仅适用于专业的科学和医学研究人员进行使用和解读，不包含任何临床建议。
本报告内容可能涉及仍处于临床研究阶段的潜在药物或靶点，这种情况下会明确标出。
Copyright 北京泛生子基因科技有限公司 2020 Ref: Genetron P2003310111

药物名称	呋喹替尼
FDA 批准适应症	（NMPA 批准的适应症）单药适用于既往接受过氟尿嘧啶类、奥沙利铂和伊立替康为基础的化疗，以及既往接受过或不适合接受抗血管内皮生长因子（VEGF）治疗、抗表皮生长因子受体（EGFR）治疗（RAS 野生型）的转移性结直肠癌（mCRC）患者。
剂型	口服常释剂型
医保支付标准	94.5 元（1mg/粒）；378 元（5mg/粒）
限定支付范围	限转移性结直肠癌患者的三线治疗。

药物名称	厄洛替尼
FDA 批准适应症	FDA 批准厄洛替尼（Erlotinib）用于携带 EGFR19 号外显子缺失或者 21 号外显子 Leu858Arg 突变的转移性非小细胞肺癌患者的一线治疗；经过四轮铂类药物化疗之后没有进展的局部晚期或转移性非小细胞肺癌的维持治疗；用于一轮铂类药物化疗失败后的局部进展或者转移性非小细胞肺癌患者；和吉西他滨联合用于局部晚期、不可切除的转移性胰腺癌一线治疗。
剂型	口服常释剂型
医保支付标准	195 元（150mg/片）<br>142.97 元（100mg/片）
限定支付范围	限 *EGFR* 基因敏感突变的晚期非小细胞肺癌。

药物名称	尼洛替尼
FDA 批准适应症	FDA 批准尼洛替尼（nilotinib）用于（1）有费城染色体阳性慢性粒性白血病(Ph+ CML)慢性期新诊断的成年患者的治疗；（2）在成年患者对既往治疗包括伊马替尼耐药或不能耐受慢性期(CP)和加速期(AP)Ph+ CML 的治疗。
剂型	口服常释剂型
医保支付标准	94.7 元（200mg/粒);

报告的内容仅适用于专业的科学和医学研究人员进行使用和解读，不包含任何临床建议。
本报告内容可能涉及仍处于临床研究阶段的潜在药物或靶点，这种情况下会明确标出。
Copyright 北京泛生子基因科技有限公司 2020 Ref: Genetron P2003310111

	76 元（150mg/粒）
限定支付范围	限治疗新诊断的费城染色体阳性的慢性髓性白血病(Ph+ CML)慢性期成人患者，或对既往治疗(包括伊马替尼)耐药或不耐受的费城染色体阳性的慢性髓性白血病(Ph+ CML)慢性期或加速期成人患者。

药物名称	芦可替尼
FDA 批准适应症	FDA 批准芦可替尼（Ruxolitinib）用于：<br>中度或高危骨髓纤维化，包括原发性骨髓纤维化、真性红细胞增多症继发的骨髓纤维化和原发性血小板增多症继发的骨髓纤维化。<br>对羟基脲反应不充分或不能耐受的成年人真性红细胞增多症。<br>12 岁及以上成人和儿童患者中类固醇难治性急性移植物抗宿主病。
剂型	口服常释剂型
医保支付标准	暂无
限定支付范围	限中危或高危的原发性骨髓纤维化（PMF）、真性红细胞增多症继发的骨髓纤维化（PPV-MF）或原发性血小板增多症继发的骨髓纤维化（PET-MF）的患者。

药物名称	培唑帕尼
FDA 批准适应症	FDA 已批准培唑帕尼（Pazopanib）用于晚期肾细胞癌患者和既往接受化疗的晚期软组织肉瘤患者的治疗。
剂型	口服常释剂型
医保支付标准	272 元（400mg/片);<br>160 元（200mg/片）
限定支付范围	晚期肾细胞癌患者的一线治疗和曾经接受过细胞因子治疗的晚期肾细胞癌的治疗。

药物名称	瑞戈非尼
FDA 批准适应症	FDA 已批准瑞戈非尼（Regorafenib）用于：

报告的内容仅适用于专业的科学和医学研究人员进行使用和解读，不包含任何临床建议。
本报告内容可能涉及仍处于临床研究阶段的潜在药物或靶点，这种情况下会明确标出。
Copyright 北京泛生子基因科技有限公司 2020 Ref: Genetron P2003310111

	（1）用于治疗既往接受过氟尿嘧啶（fluoropyrimidine），奥沙利铂（oxaliplatin）和伊立替康（irinotecan）为基础的化疗，VEGF 抑制剂治疗以及 KRAS 基因为野生型时接受过 EGFR 抑制剂治疗的转移性结直肠癌患者。<br>（2）用于治疗既往接受过伊马替尼、舒尼替尼的晚期不可切除的或转移的胃肠道间质瘤患者。<br>（3）用于治疗既往接受过索拉非尼治疗的肝细胞癌患者。
剂型	口服常释剂型
医保支付标准	196 元（40mg/片）
限定支付范围	1.肝细胞癌二线治疗；<br>2.转移性结直肠癌三线治疗；<br>3.胃肠道间质瘤三线治疗。

药物名称	塞瑞替尼
FDA 批准适应症	FDA 批准塞瑞替尼（Ceritinib）用于间变性淋巴瘤激酶（ALK）阳性转移性非小细胞肺癌患者。
剂型	口服常释剂型
医保支付标准	198 元（150mg/粒）
限定支付范围	接受过克唑替尼治疗后进展的或者对克唑替尼不耐受的间变性淋巴瘤激酶（ALK）阳性局部晚期或转移性非小细胞肺癌（NSCLC）患者。

药物名称	索拉非尼
FDA 批准适应症	FDA 已批准索拉非尼（Sorafenib）用于晚期肾细胞癌、不可切除肝细胞癌及放射性碘治疗后复发或转移的甲状腺癌患者的治疗。
剂型	口服常释剂型
医保支付标准	203 元（0.2g/片）
限定支付范围	限以下情况方可支付：<br>1.不能手术的肾细胞癌。

报告的内容仅适用于专业的科学和医学研究人员进行使用和解读，不包含任何临床建议。
本报告内容可能涉及仍处于临床研究阶段的潜在药物或靶点，这种情况下会明确标出。
Copyright 北京泛生子基因科技有限公司 2020 Ref: Genetron P2003310111

	2.不能手术或远处转移的肝细胞癌。<br>3.放射性碘治疗无效的局部复发或转移性、分化型甲状腺癌。

药物名称	维莫非尼
FDA 批准适应症	FDA 批准维莫非尼（Vemurafenib）用于治疗携带 BRAF-V600E 突变不可切除的或转移性黑色素瘤，此外还用于治疗携带 *BRAF*-V600E 突变的 Erdheim-Chester 病。
剂型	口服常释剂型
医保支付标准	112 元（240mg/片）
限定支付范围	治疗经 NMPA 批准的检测方法确定的 *BRAF* V600 突变阳性的不可切除或转移性黑色素瘤。

药物名称	西达本胺
FDA 批准适应症	（NMPA 批准的适应症）适用于既往至少接受过一次全身化疗的复发或难治的外周 T 细胞淋巴瘤患者。
剂型	口服常释剂型
医保支付标准	343 元（5mg/片）
限定支付范围	限既往至少接受过一次全身化疗的复发或难治的外周 T 细胞淋巴瘤（PTCL）患者。

药物名称	伊布替尼
FDA 批准适应症	FDA 批准用于以下患者的治疗：（1）既往至少接受过一种治疗的套细胞淋巴瘤（MCL）患者的治疗;（2）慢性淋巴细胞白血病/小淋巴细胞淋巴瘤（CLL/SLL）患者的治疗;（3）慢性淋巴细胞白血病有 17p 缺失患者的治疗;（4）Waldenstrom 氏巨球蛋白血症患者的治疗。
剂型	口服常释剂型
医保支付标准	189 元（140mg/粒）

报告的内容仅适用于专业的科学和医学研究人员进行使用和解读，不包含任何临床建议。
本报告内容可能涉及仍处于临床研究阶段的潜在药物或靶点，这种情况下会明确标出。
Copyright 北京泛生子基因科技有限公司 2020 Ref: Genetron P2003310111

限定支付范围	1.既往至少接受过一种治疗的套细胞淋巴瘤（MCL）患者的治疗;<br>2.慢性淋巴细胞白血病/小淋巴细胞淋巴瘤（CLL/SLL）患者的治疗。

药物名称	埃克替尼
FDA 批准适应症	（NMPA 批准的适应症）适用于治疗表皮生长因子（*EGFR*）基因具有敏感突变的局部晚期或转移性非小细胞肺癌患者的一线治疗。
剂型	口服常释剂型
医保支付标准	64.05 元（0.125g/片）
限定支付范围	限 *EGFR* 基因敏感突变的晚期非小细胞肺癌患者

药物名称	吉非替尼（易瑞沙）
FDA 批准适应症	FDA 已批准吉非替尼（Gefitinib）用于携带 *EGFR*19 号外显子缺失或者 21 号外显子 Leu858Arg 突变的转移性非小细胞肺癌患者的一线治疗。
剂型	口服常释剂型
医保支付标准	49.80 元（0.250g/片，齐鲁制药），228.00 元（0.250g/片，阿斯利康）
限定支付范围	限 *EGFR* 基因敏感突变的晚期非小细胞肺癌患者。

药物名称	伊马替尼
FDA 批准适应症	FDA 已批准伊马替尼（Imatinib）用于 Kit 阳性胃肠道间质瘤、慢性髓性白血病、急性淋巴细胞白血病、慢性嗜酸粒细胞白血病患者及非恶性肿瘤的治疗。
剂型	口服常释剂型
医保支付标准	14.05 元（0.1g/粒，正大天晴），12.16 元（0.1g/片，石药集团），14.05 元（0.1g/片，江苏豪森），119.20 元（0.1g/片，德国诺华）

报告的内容仅适用于专业的科学和医学研究人员进行使用和解读，不包含任何临床建议。
本报告内容可能涉及仍处于临床研究阶段的潜在药物或靶点，这种情况下会明确标出。
Copyright 北京泛生子基因科技有限公司 2020 Ref: Genetron P2003310111

限定支付范围	限有慢性髓性白血病诊断并有费城染色体阳性的检验证据的患者；有急性淋巴细胞白血病诊断并有费城染色体阳性的检验证据的儿童患者；难治的或复发的费城染色体阳性的急性淋巴细胞白血病成人患者；胃肠间质瘤患者。

药物名称	重组人血管内皮抑制素注射液（恩度）
FDA 批准适应症	（NMPA 批准的适应症）本品联合 NP 化疗方案用于治疗初治或复治的Ⅲ/Ⅳ期非小细胞肺癌患者
剂型	注射剂
医保支付标准	630 元（15mg/2.4 × 10^5 U/3ml/支）
限定支付范围	限晚期非小细胞肺癌患者。

注：1. 支付标准等具体信息以国家相关官网公布信息为准。

2. 此小结参考国家官网，只列举了国家官网公布的相关信息，具体医保信息，各地区会有部分差异。

3. 参考资料：人力资源社会保障部关于将 36 种药品纳入国家基本医疗保险、工伤保险和生育保险药品目录乙类范围的通知 http://www.mohrss.gov.cn/gkml/zlbmxgwj/ylbx_3063/201707/t20170718_274153.html

4. 参考资料：国家医疗保障局关于将 17 种抗癌药纳入国家基本医疗保险、工伤保险和生育保险药品目录乙类范围的通知 医保发〔2018〕17 号。http://www.gov.cn/xinwen/2018-10/10/content_5328891.htm

报告的内容仅适用于专业的科学和医学研究人员进行使用和解读，不包含任何临床建议。
本报告内容可能涉及仍处于临床研究阶段的潜在药物或靶点，这种情况下会明确标出。
Copyright 北京泛生子基因科技有限公司 2020 Ref: Genetron P2003310111

三、肿瘤精准诊疗实体瘤 825 基因检测列表

1.基因点突变、插入和缺失分析列表（830 个）

A2M	ABCB4	ABL1	ABL2	ACTL6A	ACTL6B	ACVR1	ACVR1B
ACVR2A	AGO2	AIP	AJUBA	AKAP9	AKT1	AKT2	AKT3
ALB	ALK	ALOX12B	AMER1	ANGPT1	ANGPT2	ANKRD11	APC
APCDD1	APLNR	APOB	AR	ARAF	ARFRP1	ARID1A	ARID1B
ARID2	ARID5B	ASCL2	ASCL4	ASXL1	ASXL2	ATF1	ATIC
ATM	ATR	ATRX	AURKA	AURKB	AXIN1	AXIN2	AXL
B2M	B4GALT3	BABAM1	BACH1	BAI1	BAI2	BAI3	BAK1
BAP1	BARD1	BBC3	BCL10	BCL2	BCL2A1	BCL2L1	BCL2L11
BCL2L2	BCL6	BCOR	BCORL1	BCR	BIRC3	BLM	BMPR1A
BRAF	BRCA1	BRCA2	BRD4	BRD7	BRIP1	BTG1	BTG2
BTK	BUB1B	C1QA	C1R	C1S	CALR	CARD11	CARM1
CASP8	CBFB	CBL	CBLB	CBR1	CCND1	CCND2	CCND3
CCNE1	CD22	CD274	CD276	CD70	CD74	CD79A	CD79B
CDC25C	CDC42	CDC73	CDH1	CDH23	CDK12	CDK2	CDK4
CDK6	CDK8	CDKN1A	CDKN1B	CDKN1C	CDKN2A	CDKN2B	CDKN2C
CDX2	CEBPA	CENPA	CFH	CFHR1	CFHR2	CFLAR	CHD2
CHD4	CHD7	CHEK1	CHEK2	CHUK	CIC	CRBN	CREBBP
CRIPAK	CRKL	CRLF2	CROT	CSDE1	CSF1R	CSF3R	CTCF
CTLA4	CTNNA1	CTNNB1	CUL3	CUL4A	CUL4B	CXCR4	CYLD
CYP17A1	CYSLTR2	DAPK1	DAXX	DCUN1D1	DDB2	DDR1	DDR2
DDX3X	DICER1	DIS3	DIS3L2	DNAJB1	DNMT1	DNMT3A	DNMT3B
DOT1L	DPYD	DROSHA	DUSP4	DUSP6	E2F3	EDNRA	EED
EGFL7	EGFR	EGR3	EIF1AX	EIF4A2	EIF4E	ELAC2	ELANE
ELF3	EML4	EMSY	EP300	EPAS1	EPCAM	EPHA2	EPHA3
EPHA4	EPHA5	EPHA7	EPHB1	EPHB2	EPHB4	EPHB6	ERBB2
ERBB3	ERBB4	ERCC1	ERCC2	ERCC3	ERCC4	ERCC5	ERF
ERG	ERRFI1	ESR1	ETV1	ETV4	ETV5	ETV6	EWSR1
EXT1	EXT2	EZH1	EZH2	EZR	F8	FAM135B	FAM175A
FAM46C	FAM58A	FANCA	FANCB	FANCC	FANCD2	FANCE	FANCF
FANCG	FANCI	FANCL	FANCM	FAS	FAT1	FAT3	FAT4
FBXW7	FCGR1A	FCGR2A	FCGR2B	FCGR2C	FCGR3A	FCGR3B	FGA
FGF10	FGF12	FGF14	FGF19	FGF23	FGF3	FGF4	FGF6
FGF7	FGFR1	FGFR2	FGFR3	FGFR4	FH	FHIT	FLCN
FLT1	FLT3	FLT4	FNTA	FOXA1	FOXA2	FOXL2	FOXO1
FOXP1	FPGS	FRK	FUBP1	FYN	FZR1	GAB2	GABRA6
GALNT12	GATA1	GATA2	GATA3	GATA4	GATA6	GDF1	GDF15
GEN1	GID4	GJB2	GLI1	GLI3	GNA11	GNA13	GNAQ

报告的内容仅适用于专业的科学和医学研究人员进行使用和解读，不包含任何临床建议。
本报告内容可能涉及仍处于临床研究阶段的潜在药物或靶点，这种情况下会明确标出。
Copyright 北京泛生子基因科技有限公司 2020 Ref: Genetron P2003310111

GNAS	GNRHR	GPC3	GPR101	GPR124	GPS2	GREM1	GRIN2A
GRM3	GSK3B	GSTT1	H3F3A	H3F3B	H3F3C	HCK	HDAC1
HDAC2	HDAC3	HDAC4	HDAC8	HDAC9	HES1	HGF	HIF1A
HIST1H1C	HIST1H2BD	HIST1H3A	HIST1H3B	HIST1H3C	HIST1H3D	HIST1H3E	HIST1H3F
HIST1H3G	HIST1H3H	HIST1H3I	HIST1H3J	HIST2H3C	HIST2H3D	HIST3H3	HLA-A
HLA-B	HLA-C	HMBS	HNF1A	HNF4A	HOXB13	HRAS	HRH2
HSD17B3	HSD3B1	HSD3B2	HSP90AA1	HSPA4	HUWE1	ICOSLG	ID3
IDH1	IDH2	IFNAR1	IFNAR2	IFNGR1	IFNGR2	IGF1	IGF1R
IGF2	IGF2R	IKBKB	IKBKE	IKZF1	IL10	IL6R	IL6ST
IL7R	INHA	INHBA	INPP4A	INPP4B	INPPL1	INSR	IRF2
IRF4	IRS1	IRS2	ITGB2	ITK	JAK1	JAK2	JAK3
JUN	KAT6A	KDM5A	KDM5C	KDM6A	KDR	KEAP1	KEL
KIF1B	KIF5B	KIT	KLF4	KLF5	KLHL6	KMT2A	KMT2B
KMT2C	KMT2D	KMT2E	KNSTRN	KRAS	LASP1	LATS1	LATS2
LCK	LIMK1	LMO1	LRP1B	LTK	LYN	LZTR1	MACF1
MAF	MALT1	MAML1	MAP2K1	MAP2K2	MAP2K3	MAP2K4	MAP3K1
MAP3K13	MAP3K14	MAPK1	MAPK3	MAPK8	MAPK8IP1	MAPKAP1	MAX
MC1R	MCL1	MDC1	MDM2	MDM4	MECOM	MED12	MEF2B
MEN1	MERTK	MET	MGA	MITF	MKNK1	MLH1	MLH3
MPL	MRE11A	MS4A1	MSH2	MSH3	MSH4	MSH5	MSH6
MSI1	MSI2	MSR1	MST1	MST1R	MTAP	MTOR	MTUS1
MUC1	MUTYH	MYB	MYBL1	MYC	MYCL	MYCL1	MYCN
MYD88	MYH9	MYOD1	NAB2	NAT1	NAT2	NBN	NCOA3
NCOR1	NCOR2	NEGR1	NEK11	NF1	NF2	NFATC2	NFE2L1
NFE2L2	NFE2L3	NFKBIA	NKX2-1	NKX3-1	NOTCH1	NOTCH2	NOTCH3
NOTCH4	NPM1	NR3C1	NRAS	NRG1	NRG3	NSD1	NT5C2
NTHL1	NTRK1	NTRK2	NTRK3	NUBPL	NUF2	NUP93	NUTM1
P2RY8	PAK1	PAK3	PAK7	PALB2	PALLD	PARK2	PARP1
PARP2	PARP3	PARP4	PAX5	PBRM1	PCBP1	PDCD1	PDCD1LG2
PDE11A	PDE4DIP	PDGFRA	PDGFRB	PDK1	PDPK1	PGR	PHF20L1
PHF6	PHOX2B	PIGF	PIK3C2B	PIK3C2G	PIK3C3	PIK3CA	PIK3CB
PIK3CD	PIK3CG	PIK3R1	PIK3R2	PIK3R3	PIM1	PLCG1	PLCG2
PLK1	PLK2	PMAIP1	PMS1	PMS2	PNRC1	POLD1	POLE
POLH	POT1	PPARG	PPM1D	PPP2R1A	PPP2R2A	PPP4R2	PPP6C
PRDM1	PRDM14	PREX2	PRKAA1	PRKACA	PRKACB	PRKAR1A	PRKCA
PRKCB	PRKCG	PRKCI	PRKD1	PRKDC	PROKR2	PRSS1	PRSS8
PSMB1	PSMB2	PSMB5	PTCH1	PTCH2	PTEN	PTK2	PTP4A1
PTP4A3	PTPN11	PTPN13	PTPRB	PTPRD	PTPRO	PTPRS	PTPRT
QKI	RAB35	RAC1	RAC2	RAD21	RAD50	RAD51	RAD51B
RAD51C	RAD51D	RAD52	RAD54L	RAF1	RARA	RARB	RARG
RASA1	RB1	RBL1	RBM10	RECQL	RECQL4	REL	RELA
RET	RFWD2	RGPD3	RHBDF2	RHEB	RHOA	RICTOR	RIT1

报告的内容仅适用于专业的科学和医学研究人员进行使用和解读，不包含任何临床建议。
本报告内容可能涉及仍处于临床研究阶段的潜在药物或靶点，这种情况下会明确标出。
Copyright 北京泛生子基因科技有限公司 2020 Ref: Genetron P2003310111

RNASEL	RNF43	ROBO1	ROCK1	ROS1	RPA1	RPL22	RPL5
RPS14	RPS6KA3	RPS6KA4	RPS6KB1	RPS6KB2	RPTOR	RRAGC	RRAS
RRAS2	RSPO2	RTEL1	RUNX1	RUNX1T1	RUNX3	RXRA	RXRB
RXRG	RYBP	SBDS	SDC4	SDHA	SDHAF2	SDHB	SDHC
SDHD	SEMA3A	SEMA3E	SERPINB3	SERPINB4	SESN1	SESN2	SESN3
SETBP1	SETD2	SETD8	SF1	SF3B1	SGK1	SH2B3	SH2D1A
SHOC2	SHQ1	SIX1	SLAMF7	SLC34A2	SLC4A1	SLIT2	SLX4
SMAD2	SMAD3	SMAD4	SMAD7	SMARCA1	SMARCA2	SMARCA4	SMARCAL1
SMARCB1	SMARCC1	SMARCC2	SMARCD1	SMARCE1	SMC1A	SMC3	SMCHD1
SMO	SMYD3	SNCAIP	SOCS1	SOS1	SOX10	SOX17	SOX2
SOX9	SPEN	SPOP	SPRED1	SPRY4	SPTA1	SRC	SRCAP
SRD5A2	SRSF2	SSTR2	STAG2	STAT1	STAT2	STAT3	STAT4
STAT5A	STAT5B	STAT6	STK11	STK19	STK40	SUFU	SUZ12
SYK	TACC3	TAF1	TAP1	TAP2	TBL1XR1	TBX3	TCEB1
TCF12	TCF3	TCF7L2	TEK	TERC	TERT	TET1	TET2
TFG	TGFBR1	TGFBR2	THADA	TIPARP	TLR4	TMEM127	TMPRSS2
TNFAIP3	TNFRSF14	TNFRSF8	TNFSF11	TNFSF13B	TOP1	TOP2A	TOP3A
TP53	TP53BP1	TP63	TP73	TPM3	TPMT	TRAF2	TRAF7
TRPS1	TRRAP	TSC1	TSC2	TSHR	TSHZ2	TTF1	TUBA1A
TUBB	TUBD1	TUBE1	TUBG1	TYR	TYRO3	U2AF1	UGT1A1
UPF1	UROD	USHBP1	USP12	USP48	USP8	VEGFA	VEGFB
VEZF1	VHL	VTCN1	WAS	WEE1	WHSC1	WHSC1L1	WISP3
WNT10A	WNT10B	WNT7B	WRN	WT1	WWTR1	XIAP	XPA
XPC	XPO1	XRCC1	XRCC2	XRCC3	YAP1	YES1	ZFHX3
ZNF148	ZNF217	ZNF521	ZNF703	ZNRF3	ZRSR2		

2.基因重排分析列表（44 个）

ALK	BCL2	BCR	BRAF	BRCA1	BRCA2	C19MC	CD74
EGFR	ETV4	ETV5	ETV6	EWSR1	EZR	FGFR1	FGFR2
FGFR3	KIT	KMT2A	MET	MSH2	MYB	MYBL1	MYC
NAB2	NOTCH2	NRG1	NTRK1	NTRK2	NTRK3	NUTM1	PDGFRA
PRKACA	RAF1	RARA	RELA	RET	ROS1	RSPO2	SDC4
SLC34A2	TERT	TMPRSS2	YAP1				

3.基因拷贝数变异分析列表（88 个）

ABL1	AKT1	AKT2	ALK	AR	ASCL2	BCL2	BRAF
C19MC	CBL	CCND1	CCNE1	CDK4	CDK6	CDK8	CSF1R
CTNNB1	DNMT3A	EGFR	ERBB2	ERBB3	ERBB4	EZH2	FGF19

报告的内容仅适用于专业的科学和医学研究人员进行使用和解读，不包含任何临床建议。
本报告内容可能涉及仍处于临床研究阶段的潜在药物或靶点，这种情况下会明确标出。
Copyright 北京泛生子基因科技有限公司 2020 Ref: Genetron P2003310111

FGF3	*FGF4*	*FGFR1*	*FGFR2*	*FGFR3*	*FGFR4*	*FLT3*	*FOXL2*
GATA2	*GNA11*	*GNAQ*	*GNAS*	*H3F3A*	*HGF*	*HNF4A*	*HRAS*
IDH1	*IDH2*	*IGF1R*	*IGF2R*	*JAK1*	*JAK2*	*JAK3*	*KDR*
KIT	*KMT2A*	*KRAS*	*MAML1*	*MAP2K1*	*MDM2*	*MDM4*	*MED12*
MET	*MPL*	*MYB*	*MYC*	*MYCL*	*MYCN*	*MYD88*	*NCOA3*
NKX2-1	*NOTCH1*	*NOTCH2*	*NOTCH3*	*NOTCH4*	*NRAS*	*PAX5*	*PDGFRA*
PIK3CA	*PTPN11*	*RET*	*RICTOR*	*RUNX1*	*SDHA*	*SERPINB3*	*SERPINB4*
SF3B1	*SMO*	*TGFBR1*	*TOP3A*	*TSHR*	*USP12*	*VEGFA*	*WHSC1L1*

4.化疗药物相关单核甘酸多态性位点（45 个）

ABCB1	rs1045642	*ABCC4*	rs9561778	*ATM*	rs1801516	*C8orf34*	rs1517114
CBR3	rs1056892	*CDA*	rs2072671	*CDA*	rs60369023	*CYP2B6*	rs3211371
DHFR	rs442767	*DPYD*	rs2297595	*DPYD*	rs3918290	*DPYD*	rs55886062
DPYD	rs67376798	*DPYD*	rs115232898	*DPYD*	rs17376848	*DPYD*	rs1801158
DPYD	rs1801159	*DPYD*	rs1801160	*DPYD*	rs1801265	*DPYD*	rs56038477
DYNC2H1	rs716274	*EGFR*	rs2293347	*EPHA5*	rs7349683	*ERBB2*	rs1136201
ERBB3	rs2229046	*ERBB3*	rs773123	*ERCC1*	rs11615	*ERCC1*	rs3212986
ERCC2	rs1052555	*ERCC2*	rs13181	*GGH*	rs11545078	*GSTP1*	rs1695
MTHFR	rs1801131	*MTHFR*	rs1801133	*MTR*	rs1805087	*NAT2*	rs1799931
NAT2	rs1801280	*SLC22A16*	rs12210538	*SOD2*	rs4880	*TP53*	rs1042522
UGT1A1	rs8175347	*UMPS*	rs1801019	*VEGFA*	rs25648	*XPC*	rs2228001
XRCC1	rs25487						

5.癌症遗传易感基因列表（148 个）

AIP	*AKT1*	*ALK*	*AMER1*	*APC*	*ATM*	*ATR*	*AXIN2*
BAP1	*BARD1*	*BLM*	*BMPR1A*	*BRCA1*	*BRCA2*	*BRIP1*	*BUB1B*
CBL	*CDC73*	*CDH1*	*CDK4*	*CDKN1B*	*CDKN1C*	*CDKN2A*	*CEBPA*
CHEK1	*CHEK2*	*CTNNB1*	*CYLD*	*DDB2*	*DICER1*	*DIS3L2*	*EGFR*
ELANE	*EPCAM*	*ERCC1*	*ERCC2*	*ERCC3*	*ERCC4*	*ERCC5*	*EXT1*
EXT2	*EZH2*	*FAM175A*	*FANCA*	*FANCB*	*FANCC*	*FANCD2*	*FANCE*
FANCF	*FANCG*	*FANCI*	*FANCL*	*FANCM*	*FAS*	*FH*	*FLCN*
GALNT12	*GATA2*	*GEN1*	*GPC3*	*GREM1*	*HMBS*	*HNF1A*	*HOXB13*
HRAS	*KIT*	*LASP1*	*MAX*	*MC1R*	*MEN1*	*MET*	*MITF*
MLH1	*MLH3*	*MRE11A*	*MSH2*	*MSH3*	*MSH6*	*MTAP*	*MTUS1*
MUTYH	*NBN*	*NF1*	*NF2*	*NSD1*	*NTHL1*	*NTRK1*	*PALB2*
PALLD	*PDE11A*	*PDGFRA*	*PHOX2B*	*PMS1*	*PMS2*	*POLD1*	*POLE*
POLH	*PPM1D*	*PRKAR1A*	*PRSS1*	*PTCH1*	*PTCH2*	*PTEN*	*PTPN11*
RAD50	*RAD51*	*RAD51B*	*RAD51C*	*RAD51D*	*RB1*	*RECQL*	*RECQL4*

报告的内容仅适用于专业的科学和医学研究人员进行使用和解读，不包含任何临床建议。
本报告内容可能涉及仍处于临床研究阶段的潜在药物或靶点，这种情况下会明确标出。
Copyright 北京泛生子基因科技有限公司 2020 Ref: Genetron P2003310111

RET	*RHBDF2*	*RUNX1*	*RUNX3*	*SBDS*	*SDHA*	*SDHAF2*	*SDHB*
SDHC	*SDHD*	*SLX4*	*SMAD4*	*SMARCA4*	*SMARCB1*	*SMARCE1*	*SOS1*
STAT3	*STK11*	*SUFU*	*TERT*	*TGFBR1*	*TGFBR2*	*TMEM127*	*TP53*
TP63	*TSC1*	*TSC2*	*UROD*	*USHBP1*	*VEGFA*	*VHL*	*WRN*
WT1	*XPA*	*XPC*	*XRCC2*				

报告的内容仅适用于专业的科学和医学研究人员进行使用和解读，不包含任何临床建议。
本报告内容可能涉及仍处于临床研究阶段的潜在药物或靶点，这种情况下会明确标出。
Copyright 北京泛生子基因科技有限公司 2020 Ref: Genetron P2003310111

四、样本主要质控

	质量参数	数值	质控标准
病理评估	恶性肿瘤细胞占比(%)[1]	55	≥10%
DNA 质量评估	DNA 总量(ng)[2]	1970	≥100ng
测序质量评估	平均测序深度 [3]	1718.23	≥500X
	≥140X 深度位点占比 [4]	99.41%	≥90%
	捕获效率 [5]	61.90%	≥45%
	GC 含量 [6]	47.50%	40%-60%
	碱基质量 Q30 占比 [7]	97.70%	≥80%
总体质量评估 [8]		合格	

注：1. 恶性肿瘤细胞占比：经泛生子医学检验所HE染色评估，该样本中恶性肿瘤细胞占比。如样本不满足泛生子医学病理评估所需条件，则跳过此项。血浆cfDNA样本不做此项评估。

2. DNA总量：送检样本提取的DNA总量。

3. 平均测序深度：目标基因每个碱基被测到的平均次数。

4. ≥140X深度位点占比：测序深度超过140X的位点占所有目标检测位点的比例，可评价测序均一性。

5. 捕获效率：目标区域序列数与测定的总序列的比值

6．GC含量：在DNA 4种碱基中，鸟嘌呤（G）和胞嘧啶（C）所占的比率。

7. 碱基质量Q30占比：测序数据中碱基质量在Q30以上（即错误率在千分之一以下）的占比。

8. 总体质量评估：结合以上参数进行综合评估，采取短板效应，分为合格、警戒（风险预警）和不合格三个等级。质量警戒或不合格都可能会影响此次检测的准确性和敏感性。

报告的内容仅适用于专业的科学和医学研究人员进行使用和解读，不包含任何临床建议。
本报告内容可能涉及仍处于临床研究阶段的潜在药物或靶点，这种情况下会明确标出。
Copyright 北京泛生子基因科技有限公司 2020 Ref: Genetron P2003310111

【本页为报告签字盖章页，无报告正文】

检测人：

审核人：

北京泛生子基因科技有限公司
报告专用章

报告的内容仅适用于专业的科学和医学研究人员进行使用和解读，不包含任何临床建议。
本报告内容可能涉及仍处于临床研究阶段的潜在药物或靶点，这种情况下会明确标出。
Copyright 北京泛生子基因科技有限公司 2020 Ref: Genetron P2003310111

品质保证–全周期质控体系

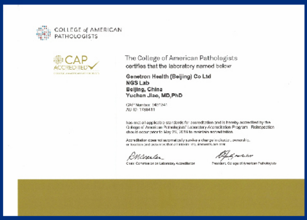

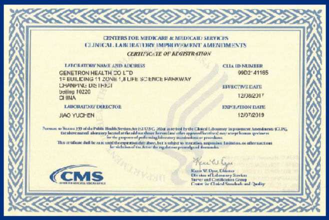

泛生子一次性通过 CAP、CLIA 的双认证

CAP – 美国病理学家协会：COLLEGE OF AMERICAN PATHOLOGISTS
CLIA – 临床检验改进修正计划：Clinical Laboratory Improvement Amendments

泛生子建立了严苛的质控体系，以最大程度确保实验室检测结果的准确性、可靠性和时效性达到国际领先水平，获得国际权威机构认可。

6 个质控节点： 样本复核、肿瘤纯度评估、核酸质检、文库质检、下机数据质控、报告复核。

5 大维度把控： 对实验室的人员、仪器、耗材、方法、环境进行系统的规范和管理。

3 套质控策略： 贯穿检测全过程的室内质量控制、清晰落地的 SOP 以及定期参加权威机构的室间质评。

genetronhealth.com

T: 010-50907500
M: customer@genetronhealth.com
北京市昌平区中关村
生命科学园生命园路8号院1区11号楼
