# Supplementary material for: Choroid Plexus Carcinomas With TP53 Germline Mutations: Management and Outcome
Source: Front Oncol. 2021 Sep 30;11:751784. doi: 10.3389/fonc.2021.751784 (PMC8514937; doi:10.3389/fonc.2021.751784)
Supplement: Supplementary file 1 [file DataSheet_1.zip › original_data/Θ¡Åσ¡Éτæ£_F20100002T_P2009290064_fwa-glioma-6.pdf]

GENETRON 泛生子

洞悉癌症全周期

肿瘤精准诊疗基因检测

脑肿瘤分子检测报告（六项）

ANSWERS
FOR
CANCER

目 录

检测结果……2

检测项目的意义解析……3

分子检测方法介绍……4

本报告结果仅针对本次送检样品，结果仅供专业人士参考，不包含任何临床建议。

报告编号：P2009290064-20201006115841

受检者 姓 名	魏子瑜	样 本 编 号	F20100002T
年 龄	0	样本采集日期	2020-09-29
性 别	男	样本接收日期	2020-10-01
样 本 类 型	手术组织石蜡包埋切片	出具报告日期	2020-10-06

脑肿瘤分子检测报告（六项）

检测结果

检测项目	检测结果
MGMT 启动子甲基化	无甲基化
1p 染色体杂合性缺失	完整
19q 染色体杂合性缺失	缺失
IDH1 基因 R132 突变	无突变
IDH2 基因 R172 突变	无突变
TERT 基因 C228T 突变	无突变
TERT 基因 C250T 突变	无突变
BRAF 基因 V600E 突变	无突变

提示：

若 1p 染色体与 19q 染色体同时检测出杂合性缺失则为联合缺失，若仅一项或两项都未检测出缺失则为非联合缺失。

MGMT 启动子甲基化具体检测结果见分子检测方法及结果解析部分。

结果解读

1. *MGMT* 启动子甲基化倾向于在替莫唑胺治疗中获益。

2. 1p19q 联合缺失与 *IDH* 突变提示病理分型为少突胶质细胞瘤，生存期较长，对放、化疗敏感[1,2]。

3. *IDH1* 或 *IDH2* 基因突变多存在于低级别胶质瘤，亦见于继发性胶母细胞瘤。*IDH1* 或 *IDH2* 中任一基因突变提示低风险，预后优于相同级别相同亚型无突变的胶质瘤[1,2]。

4. *TERT* 和 *IDH* 基因突变联合分析可辅助判断预后不同的分子分型[3,4]。

5. 维莫非尼在携带 *BRAF* 突变的儿童胶质母细胞瘤、毛细胞黏液样星形细胞瘤等的治疗中均取得了较好的疗效[5,6]；携带 *BRAF* 基因 V600E 突变的黑色素瘤患者可能在维莫非尼、达拉非尼治疗中获益[7,8]。

检测方法

肿瘤基因组 DNA 从肿瘤组织中提取纯化后，经过质检和初步定量，制定最佳检测方案进行相应分子检测。

IDH/TERT/BRAF 基因突变和 1p19q 杂合性缺失使用一步法检测；*MGMT* 甲基化检测使用焦磷酸测序法。

本报告结果仅针对本次送检样品，结果仅供专业人士参考，不包含任何临床建议。

检测项目的意义解析

生物标志物[1,9]

分子标志物	生物学功能	诊断价值	预后价值	预测价值
*IDH*1/2 突变	增加与 G-CIMP 亚型相关的 2-泾戊二酸的浓度	胶质瘤分型	相比无突变的患者，存在 *IDH*1/2 突变的患者预后较好	如无突变、建议检测 *MGMT* 启动子甲基化来预测预后
lp/19q 联合性缺失	1p19q 指 1 号染色体短臂和 19 号染色体长臂，1p/19q 杂合缺失是由于非平衡异位引起。	与少突胶质细胞瘤密切相关，与其他具有透明细胞的脑肿瘤区分	有联合性缺失的患者预后较好	对于有联合缺失的少突或间变性少突胶质细胞瘤患者、推荐化疗或联合放、化疗
MGMT 启动子甲基化	干扰 DNA 修复、与 *IDH* 1/2 突变肿瘤中 G-CIMP 相关	无	对于间变性胶质瘤患者(可能伴有 *IDH* 突变)放/化疗有好的疗效	有 *MGMT* 启动子甲基化的 GBM(可能没有 *IDH* 突变）对烷化剂敏感。对老年患者有预测价值。
TERT 基因启动突变	对端粒长度维持有关键作用，突变时肿瘤中端粒酶异常表达的重要机制之一	胶质瘤分型	在原发性胶母细胞瘤中不与 IDH 突变同时存在，提示预后不良；而少突胶质细胞瘤常表现为 *TERT* 基因启动子与 *IDH1*/2 基因双突变，提示良好的预后。	联合检测 *TERT* 启动子突变和 *IDH* 1/2 突变可用于弥散型的分子分型和预后判断（II 级证据）
BRAF 点突变	激活 MAPK 信号转导通路	胶质瘤的诊断及未来靶向药物的治疗	不明确	*BRAF* 基因检测可协助儿童或青少年胶质瘤的诊断和指导未来的靶向治疗

本报告结果仅针对本次送检样品，结果仅供专业人士参考，不包含任何临床建议。

分子检测方法及结果解析

1. *MGMT* 基因启动子甲基化检测

1.1 *MGMT* 基因启动子 CpG 岛甲基化在判断脑胶质瘤患者预后及预测肿瘤对烷化剂药物耐药性方面具有重要意义。*MGMT* 基因启动子甲基化倾向于在替莫唑胺治疗中获益。MGMT 启动子甲基化检测主要针对位于 10 号染色体上 4 个 CpG 位点。结果通过判断 4 个 CpG 位点的甲基化比列均值获得，当均值大于 10%时，结果判定为阳性，否则为阴性。

1.2 焦磷酸测序法能够高度精确地分析一个或多个 CpG 位点的甲基化序列单个位点的变化。检测仪器：Qiagen 公司 PyroMark Q24 焦磷酸测序仪，PyroMark Q24 包含了一个完整软件包，用于分析 CpG 位点甲基化，以及用于确认亚硫酸氢盐处理彻底与否的内置对照。

Position	LOB (% units)
CpG site 1	1.5
CpG site 2	1.8
CpG site 3	3.2
CpG site 4	3.4
Mean of CpG site 1 to 4	2.1

Table 1. LOB determined for specific methylation sites using samples from healthy blood donors

***MGMT* 基因启动子甲基化检测结果：**

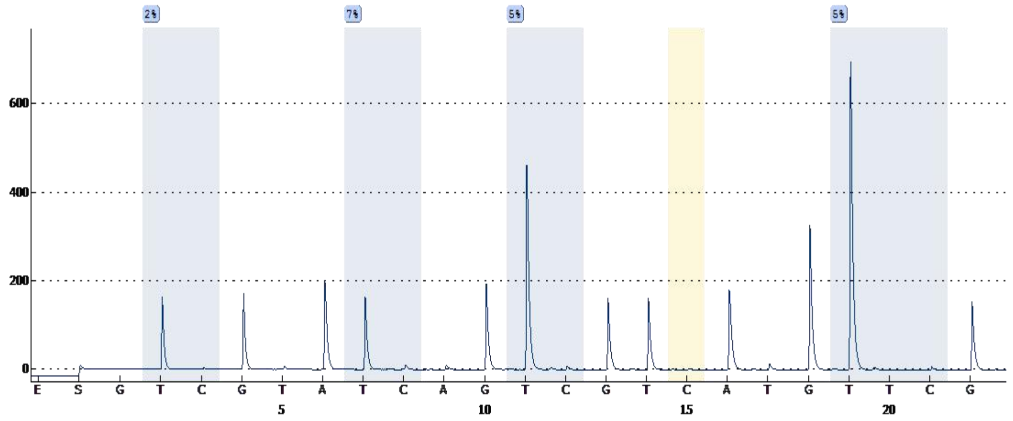

本次检测样品 *MGMT* 基因启动子区甲基化比例为 4.750%。

本报告结果仅针对本次送检样品，结果仅供专业人士参考，不包含任何临床建议。

***MGMT* 基因启动子甲基化检测结果解释**

1）阳性

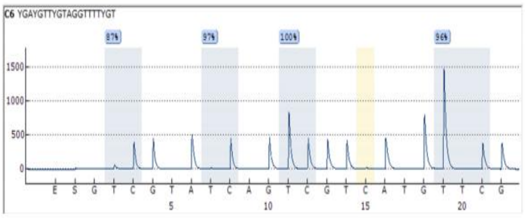

图 1：*MGMT* CpG site1 - 4 甲基化比例

阳性结果显示为：甲基化

（site1：87%；site2：97%；

site3：100%；site4：96%）

2）阴性

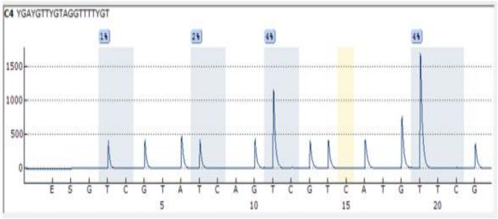

图 2：*MGMT* CpG site1 - 4 甲基化比例

阴性结果显示为：无甲基化

（site1：1%；site2：2%；

site3：4%；site4：4%）

2. 1p19q 杂合性缺失检测

2.1 1 号染色体短臂末端集中了高密度的与细胞生长、增殖、分化相关的基因，所以这一区域成为肿瘤分子细胞遗传研究的热点，尤其是在神经母细胞瘤、结肠癌和乳腺癌的研究领域。在 1p/19q 的热点区的微卫星序列上设计引物可以检测到是否能扩增出相应区段，从而检测缺失情况。

2.2 检测方法：一步法（多重 PCR 扩增联合 NGS 测序）。检测位点经过多重 PCR 扩增后，取适量 PCR 产物利用 Ion Torrent Proton 平台测序，并对测序结果进行分析。多重 PCR(multiplex PCR)，又称多重引物 PCR 或复合 PCR，它是在同一 PCR 反应体系里加上两对以上引物，同时扩增出多个核酸片段的 PCR 反应。

2.3 检测位点：1p 和 19q 染色体杂合性缺失

3. *TERT* 基因启动子突变检测

3.1 端粒酶逆转录酶（Telomerase Reverse Transcriptase，*TERT*）是端粒酶复合物的催化中心，*TERT* 基因启动子区突变可以增加端粒酶的活性，与肿瘤细胞旺盛的生长能力和无限分裂有关。最新研究表明：只携带 *TERT* 突变的 III-IV 级胶质瘤患者多为原发性胶母细胞瘤，且预后不良；只携带 *IDH1*/2 突变的 III-IV 级胶质瘤患者多呈现星形细胞形态；同时携带 *TERT* 和 *IDH1*/2 突变的胶质瘤患者多呈现少突胶质细胞形态，预后良好。

3.2 检测方法：一步法（多重 PCR 扩增联合 NGS 测序）

3.3 检测位点：*TERT*（C228T、C250T）

4. *IDH*1 &*IDH*2 基因突变检测

4.1 异柠檬酸脱氢酶同工酶 1（*IDH1*）基因位于 2 号染色体长臂(2q33)，编码异柠檬酸脱氢酶同工酶 1，催化异柠檬酸盐氧化羧化成α-酮戊二酸，生成三羧酸循环中的 NADPH。人体共有 5 种 *IDH* 基因编码 3 种不同的 *IDH* 酶，分别为 *IDH1*、*IDH2* 和 *IDH3* 酶。*IDH1* 和 *IDH2* 突变造成另一种代谢产物——2-羟戊二酸在细胞中积累，既导致组蛋白修饰异常，也能使 VEGF 通路活性增高，从而诱发肿瘤发生。美国国家综合癌症网络（NCCN）临床实践指南将有无 *IDH1*/2 基因突变作为评估低级别胶质瘤患者风险级别的指标之一[10]。另外，*IDH1* 突变在恶性髓系疾病中的临床及生物学特征已陆续被报道。

本报告结果仅针对本次送检样品，结果仅供专业人士参考，不包含任何临床建议。

4.2 检测方法：一步法（多重 PCR 扩增联合 NGS 测序）

4.3 检测位点：

基因	检测位点				
IDH1	R132H	R132C	R132S	R132G	R132L
IDH2	R172K	R172M	R172W	R172G	R172S

5. *BRAF* 基因突变检测

5.1 *BRAF* 基因在多种肿瘤中存在突变，最为典型的是第 600 位氨基酸缬氨酸突变为谷氨酸 (V600E)，该位点的突变导致 *BRAF* 激酶异常激活，并持续激活下游 MEK-ERK 信号通路，促进肿瘤细胞的生长增殖和转移。 近年临床试验表明，维莫非尼在儿童胶质母细胞瘤、毛细胞黏液样星形细胞瘤、复发多形性星形细胞瘤等的治疗中均也取得了较好的疗效，提示 *BRAF* 突变的患者可选取维莫非尼可作为潜在靶向的治药物。

5.2 检测方法：一步法（多重 PCR 扩增联合 NGS 测序）

5.3 检测位点：*BRAF* V600E

本报告结果仅针对本次送检样品，结果仅供专业人士参考，不包含任何临床建议。

参考文献 [References]

[1] WHO Classification of Tumours of the Central Nervous System. Version 4.2016.

[2] High-grade glioma：ESMO Clinical Practice Guidelines for diagnosis, treatment and follow-up. Annals of Oncology 00:1-9, 2014.

[3] Killela PJ, Yan H, Bigner DD, et al. Mutations in IDH1, IDH2, and in the TERT promoter define clinically distinct subgroups of adult malignant gliomas. Oncotarget 2014; 5(6):1515-1525.

[4] M Labussiere, A L Di Stefano, M Sanson, et al. TERT promoter mutations in gliomas, genetic associations and clinico-pathological correlations. Br J Cancer 2014; 111:2024-2032.

[5] Robinson G W, Orr B A, Gajjar A. Completeclinical regression of a BRAF V600E-mutant pediatric glioblastoma multiformeafter BRAF inhibitor therapy [J]. BMC cancer, 2014,14(1):258.

[6] Skrypek M, Foreman N, Guillaume D, et al. Pilomyxoid astrocytoma treated successfully with vemurafenib[J]. Pediatricblood& cancer, 2014, 61(11):2099-2100.

[7] Chapman P B, Hauschild A, Robert C et al. Improved survivalwithvemurafenib in melanoma with BRAF V600E mutation [J]. New England Journalof Medicine, 2011, 364(26): 2507-2516.

[8] Luke J J ,HodiF S. Ipilimumab, vemurafenib, daBRAFenib, and trametinib: synergisticcompetitors in the clinical management of BRAF Mutant malignant melanoma [J]. The oncologist, 2013, 18(6):717-725.

[9] Chin J Neurosurg , May 2014, V0L 30, No,5；中国脑胶质瘤分子诊疗指南，-临床指南.

[10] Sanson, M. et al. Isocitrate dehydrogenase 1codon 132 mutation is an important prognosticbiomarker in gliomas. J. Clin. Oncol.27,4150–4154 (2009).

声明

对有限数量的细胞进行基因检测不能揭示所有可能存在的有意义的基因突变，也不能排除肿瘤进展过程中发生变化的可能性。基因突变可能由于检测技术的限制、标本中肿瘤细胞比例、样本采集或运输意外以及其他无法预知大因素等而未被发现。本报告仅对检测样本和检测报告的一致性负责。

本报告结果仅针对本次送检样品，结果仅供专业人士参考，不包含任何临床建议。

【本页为报告签字盖章页，无报告正文】

检测人：　　　　审核人：

北京泛生子基因科技有限公司

报告专用章

本报告结果仅针对本次送检样品，结果仅供专业人士参考，不包含任何临床建议。

品质保证-全周期质控体系

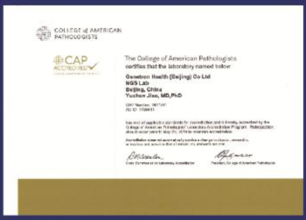

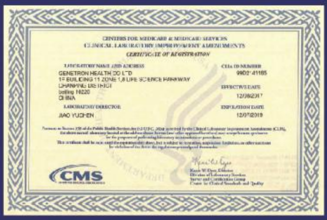

泛生子一次性通过 CAP、CLIA 的双认证

CAP – 美国病理学家协会：COLLEGE OF AMERICAN PATHOLOGISTS
CLIA – 临床检验改进修正计划：Clinical Laboratory Improvement Amendments

泛生子建立了严苛的质控体系，以最大程度确保实验室检测结果的准确性、可靠性和时效性达到国际领先水平，获得国际权威机构认可。

6 个质控节点： 样本复核、肿瘤纯度评估、核酸质检、文库质检、下机数据质控、报告复核。

5 大维度把控： 对实验室的人员、仪器、耗材、方法、环境进行系统的规范和管理。

3 套质控策略： 贯穿检测全过程的室内质量控制、清晰落地的 SOP 以及定期参加权威机构的室间质评。

genetronhealth.com

T: 010-50907500
M: customer@genetronhealth.com
北京市昌平区中关村
生命科学园生命园路8号院1区11号楼
