# Supplementary material for: Choroid Plexus Carcinomas With TP53 Germline Mutations: Management and Outcome
Source: Front Oncol. 2021 Sep 30;11:751784. doi: 10.3389/fonc.2021.751784 (PMC8514937; doi:10.3389/fonc.2021.751784)
Supplement: Supplementary file 1 [file DataSheet_1.zip › original_data/F20124115T_σ«üΣ╕ÇΦ»║.pdf]

免疫组化PD-L1检测咨询结果

1、基本信息

**患者姓名：**	宁一诺	**样本编号：**	F20124115T
**终端客户编号：**	P2012220286	**样本类型：**	手术组织石蜡包埋切片
**性别：**	男	**样本采集部位：**	
**年龄：**	3岁	**样本采集日期：**	2020-12-22
**既往诊断结果：**	脉络丛乳头状癌，WHO III级	**样本接收日期：**	2020-12-24
**既往治疗方案：**		**报告日期：**	2020-12-28

2、镜下所见（仅供参考）

· 质控样本结果：

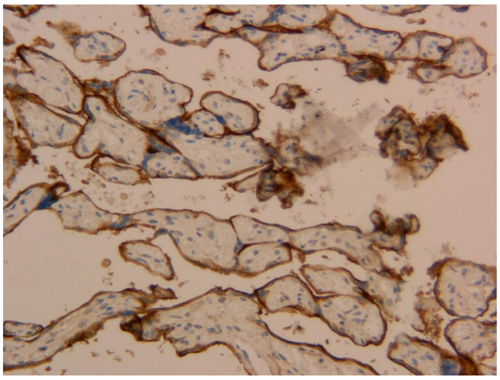

阳性质控片

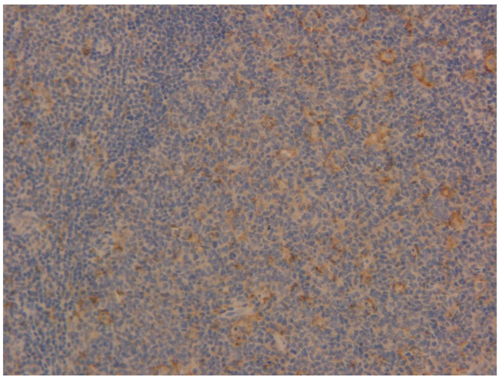

阴性质控片

· 检测样本结果：

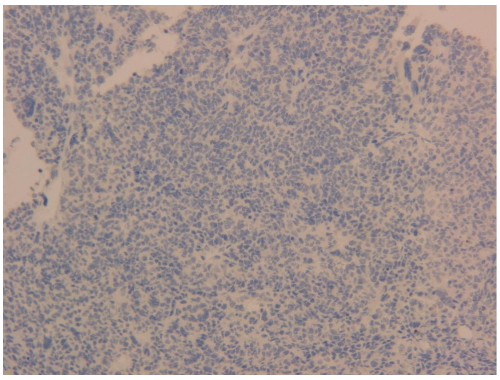

IHC

备注：本报告仅对本次标本检测负责，结果仅供医生参考。

Copyright 北京泛生子基因科技有限公司

网址：www.genetronhealth.com 客服电话：010-50907500
地址：北京市昌平区中关村生命科学园生命园路 8 号院 1 区 11 号楼 1/2 层

3、检测结果

检测蛋白	抗体型号	检测方法	检测结果
PD-L1	22C3	IHC	TPS<1%

注：

（1） 本检测结果仅对所检测样本负责；

（2） 本结果可能对部分癌种免疫药物疗效有一定的指导作用，但具体治疗方案需由医生根据病人病史/用药史决定，本报告仅做参考。

结果解析参考：

① TPS： 在任何强度下显示部分或完全膜染色的活肿瘤细胞的百分比。目前 FDA 推荐肺癌等癌种在使用 Pembrolizumab 时，通过检测 TPS 评价 PD-L1 表达情况。

表1：PD-L1 TPS表达阈值划分

Tumor Proportion Score(TPS)			
PD-L1 Expression Levels	TPS<1%	TPS=1-49%	TPS≥50%
PD-L1 Expression Status	No PD-L1 Expression	PD-L1 Expression	High PD-L1 Expression

注：界值划分参考 PD-L1 IHC 22C3 dx 获批说明书

表2：PD-L1表达与Pembrolizumab疗效（肺癌）

临床研究名称	药物	PD-L1 TPS	入组患者数	ORR,% Pem vs Doc	中位 OS,月 Pem vs Doc	中位 PFS,月 Pem vs Doc
KEYNOTE-010	Pembrolizumab vs Docetaxel(Doc)	1-24%	471	8.6 vs 10.9	9.7 vs 8.5	2.6 vs 4.0
		25-49%	120	15.8 vs 9.1	9.8 vs 9.9	2.9 vs 3.8
		50-74%	158	22.6 vs 9.6	15.8 vs 8.2	4.3 vs 4.3
		≥75%	284	33.7 vs 7	16.2 vs 8.2	6.2 vs 4.0

注：参考肺癌 KEYNOTE-010 临床研究

Copyright 北京泛生子基因科技有限公司

② CPS： PD-L1染色细胞（肿瘤细胞、淋巴细胞、巨噬细胞）的数目除以活的肿瘤细胞总数乘以100。目前FDA推荐胃癌/胃食管交界处腺癌、食管鳞状细胞癌、尿路上皮癌、宫颈癌、头颈部鳞状细胞癌等癌种在使用 Pembrolizumab 时，通过检测 CPS 评价 PD-L1 表达情况。

表3：PD-L1 CPS表达阈值划分

Combined Positive Score(CPS)		
PD-L1 Expression Levels	CPS<1	CPS≥1
PD-L1 Expression Status	No PD-L1 Expression	PD-L1 Expression

注：界值划分参考 PD-L1 IHC 22C3 dx 获批说明书

表4：PD-L1表达与Pembarolizumab疗效（胃癌/胃食管交接腺癌）

临床研究名称	药物	PD-L1 CPS	入组患者数	ORR,%	CR,%	PR,%
KEYNOTE-059	Pembrolizumab	≥1	143	13.3	1.4	11.9

注：参考胃癌 KEYNOTE-059 临床研究

表5：PD-L1表达与Pembrolizumab疗效（食管鳞状细胞癌）

临床研究名称	药物	PD-L1 CPS	入组患者数	ORR,%	CR,%	PR,%
KEYNOTE-181	Pembrolizumab	≥10	79	21.5	3	14

注：参考食管鳞状细胞癌 KEYNOTE-181 临床研究

备注：本报告仅对本次标本检测负责，结果仅供医生参考。
Copyright 北京泛生子基因科技有限公司
网址：www.genetronhealth.com 客服电话：010-50907500
地址：北京市昌平区中关村生命科学园生命园路 8 号院 1 区 11 号楼 1/2 层

表6：PD-L1表达与Pembrolizumab疗效（尿路上皮癌）

临床研究名称	药物	PD-L1 CPS	入组患者数	ORR,%	CR,%	PR,%
KEYNOTE-052	Pembrolizumab	≥10	80	51	16	35

注：参考尿路上皮癌 KEYNOTE-052 临床研究

表7：PD-L1表达与Pembrolizumab疗效（宫颈癌）

临床研究名称	药物	PD-L1 CPS	入组患者数	ORR,%	CR,%	PR,%
KEYNOTE-158	Pembrolizumab	≥1	77	14.3	2.6	11.7

注：参考宫颈癌 KEYNOTE-158 临床研究

表8：PD-L1表达与Pembrolizumab疗效（头颈部鳞状细胞癌）

临床研究名称	药物	PD-L1 CPS	入组患者数	ORR,%	CR,%	PR,%
KEYNOTE-048	Pembrolizumab	≥1	257	19	5	14
KEYNOTE-048	Pembrolizumab	≥20	133	23	8	16

注：参考头颈部鳞状细胞癌 KEYNOTE-048 临床研究

③ 对于未获得 FDA 批准的 Dako 22C3 伴随诊断适用的癌种，使用 Pembrolizumab 时默认通过检测 TPS 评价 PD-L1 表达情况。

Copyright 北京泛生子基因科技有限公司

4、样本质控情况

质量参数	数值	质控标准
恶性肿瘤细胞个数（个）	> 100	≥100
总体质量评估	合格	

Copyright 北京泛生子基因科技有限公司
网址：www.genetronhealth.com 客服电话：010-50907500
地址：北京市昌平区中关村生命科学园生命园路 8 号院 1 区 11 号楼 1/2 层

附录：

1、PD-L1试剂盒介绍

抗体型号	诊断平台	PD1/PD-L1药物	FDA获批情况
22C3	Dako	Pembrolizumab	伴随诊断（肺癌）
28-8	Dako	Nivolumab	补充诊断（肺癌）
SP142	Ventana Benchmark	Atezolizumab	补充诊断（肺癌）
SP263	Ventana Benchmark	Durvalumab	\
73-10	Dako	Avelumab	\

2、Pembrolizumab获批癌种

药物	商品名/药企	靶点	检测抗体	FDA获批癌种	NMPA获批癌种
Pembrolizumab	Keytruda/<br>默沙东	PD-1	22C3	NSCLC<br>MSI-H/dMMR实体瘤<br>结直肠癌<br>头颈鳞癌<br>黑色素瘤<br>霍奇金淋巴瘤<br>原发性纵隔大b细胞淋巴瘤<br>尿路上皮癌<br>胃及胃食管交界处腺癌<br>宫颈癌<br>肝细胞肝癌<br>肾细胞癌<br>食管癌	黑色素瘤<br>NSCLC

Copyright 北京泛生子基因科技有限公司
网址：www.genetronhealth.com 客服电话：010-50907500
地址：北京市昌平区中关村生命科学园生命园路 8 号院 1 区 11 号楼 1/2 层
